# TGFβ2‐Driven Ferritin Degradation and Subsequent Ferroptosis Underlie Salivary Gland Dysfunction in Postmenopausal Conditions

**DOI:** 10.1002/advs.202400660

**Published:** 2024-11-01

**Authors:** Su‐Jeong Oh, Ye Young Shin, Ji‐Su Ahn, Hee‐Jeong Park, Min‐Jung Kang, Tae‐Hoon Shin, Byung‐Chul Lee, Won Kyu Kim, Jung‐Min Oh, Dongjun Lee, Yun Hak Kim, Ji Min Kim, Eui‐Suk Sung, Eun‐Woo Lee, Jee‐Heon Jeong, Byung‐Joo Lee, Yoojin Seo, Hyung‐Sik Kim

**Affiliations:** ^1^ Department of Oral Biochemistry, Dental and Life Science Institute, School of Dentistry Pusan National University Yangsan 50612 Republic of Korea; ^2^ Department of Life Science in Dentistry School of Dentistry Pusan National University Yangsan 50612 Republic of Korea; ^3^ Education and Research Team for Life Science on Dentistry Pusan National University Yangsan 50612 Republic of Korea; ^4^ Stem Cell and Regenerative Bioengineering Institute Global R&D Center Kangstem Biotech Co. Ltd. Seoul 08590 Republic of Korea; ^5^ Department of Laboratory Animal Medicine College of Veterinary Medicine and Veterinary Medical Research Institute Jeju National University Jeju‐si 63243 Republic of Korea; ^6^ Department of Biological Sciences Sookmyung Women's University Seoul 04310 Republic of Korea; ^7^ Research Institute of Women’s Health Sookmyung Women’s University Seoul 04310 Republic of Korea; ^8^ Natural Product Research Center Korea Institute of Science andTechnology (KIST) Gangneung 25451 Republic of Korea; ^9^ Department of Convergence Medicine Yonsei University Wonju College of Medicine Wonju 26426 Republic of Korea; ^10^ Division of Natural Products Applied Science University of Science and Technology (UST) Daejeon 34113 Republic of Korea; ^11^ Department of Convergence Medicine Pusan National University School of Medicine Yangsan 50612 Republic of Korea; ^12^ Department of Anatomy Pusan National University School of Medicine Yangsan 50612 Republic of Korea; ^13^ Department of Otorhinolaryngology‐Head and Neck Surgery Pusan National University School of Medicine and Biomedical Research Institute Pusan National University Hospital Busan 49241 Republic of Korea; ^14^ Department of Otorhinolaryngology‐Head and Neck Surgery Biomedical Research Institute Pusan National University School of Medicine Yangsan Pusan National University Hospital Yangsan 50612 Republic of Korea; ^15^ Metabolic Regulation Research Center Korea Research Institute of Bioscience and Biotechnology (KRIBB) Daejeon 34141 Republic of Korea; ^16^ School of Pharmacy Sungkyunkwan University Suwon 16419 Republic of Korea; ^17^ Department of Precision Medicine School of Medicine Sungkyunkwan University Suwon 16419 Republic of Korea

**Keywords:** estrogen, ferroptosis, organoids, salivary gland, TGFβ2, xerostomia

## Abstract

Despite the high incidence of dry mouth in postmenopausal women, its underlying mechanisms and therapeutic interventions remain underexplored. Using ovariectomized (OVX) mouse models, here this study identifies ferroptosis, an iron‐dependent regulated cell death, as a central mechanism driving postmenopausal salivary gland (SG) dysfunction. In the OVX‐SGs, TGFβ signaling pathway is enhanced with the aberrant TGFβ2 expression in SG mesenchymal cells. Intriguingly, TGFβ2 treatment reduces iron‐storing ferritin levels, leading to lipid peroxidation and ferroptotic death in SG epithelial organoids (SGOs). Mechanistically, TGFβ2 promotes the autophagy‐mediated ferritin degradation, so‐called ferritinophagy. A notable overexpression of the type III TGFβ receptor (TβRIII) is found in the OVX‐SGs and TGFβ2‐treated SGOs, while the silencing of TβRIII mitigates the ferroptosis‐mediated deleterious effects of TGFβ2 on SGOs. Finally, administration of ferroptosis inhibitor, Liproxstatin‐1 (Lip‐1), improves saliva secretion in OVX mice. Present findings collectively suggest a link between TGFβ signaling, ferroptosis, and SG injury, offering new therapeutic avenues for postmenopausal xerostomia.

## Graphical abstract

Ferroptosis is identified as a key mechanism in postmenopausal salivary gland dysfunction using ovariectomized (OVX) mouse models. Enhanced TGFβ signaling via TGFβ2‐TβRIII axis triggers ferritin degradation and lipid peroxidation, leading to ferroptosis in SG epithelial organoids. Administration of the ferroptosis inhibitor Liproxstatin‐1 (Lip‐1) restores SG function in OVX mice, unveiling promising therapeutic avenues for postmenopausal xerostomia.

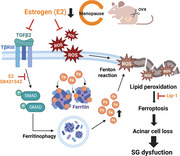

## 1 Introduction

Reduced or absent salivary flow resulting from salivary gland (SG) dysfunction causes xerostomia, a perception of aberrant oral dryness. The development of xerostomia is correlated with various factors including radiation therapy, medication (e.g., anticholinergic drugs), autoimmune disorders (e.g., Sjogren's syndrome and rheumatism), infectious agents (e.g., HIV and hepatitis C), metabolic diseases (e.g., diabetes) and physiological aging.^[^ ^1^, ^2^ ^]^ Notably, persistent xerostomia is one of the most frequent complications that increase with age and imposes detrimental impacts on the quality of life in the elderly population.^[^ ^2^ ^]^ However, due to the disease's underestimated significance and limited understanding of SG dysfunction, current xerostomia treatments primarily rely on temporary symptom‐control strategies, such as saliva substrates and pain relief—with no fundamental therapy having yet been developed.^[^ ^3^ ^]^

In general, women are more susceptible to xerostomia than men; clinical studies have indicated a noteworthy increase in the incidence of xerostomia following menopause,^[^ ^4^ ^]^ along with a negative correlation between saliva estrogen levels and the severity of xerostomia symptoms.^[^ ^5^, ^6^ ^]^ Hormonal replacement therapy has been found beneficial in restoring saliva secretion in postmenopausal women, indicating the importance of estrogen in maintaining SG function.^[^ ^6^ ^]^ Similarly, ovariectomy (OVX) in rodents not only decreases saliva secretion but also triggers pathological alterations in the SG, including fat accumulation, fibrosis, and enhanced oxidative stress.^[^ ^7^, ^8^ ^]^ In particular, we recently reported an increase in lipid peroxidation and iron deposits in rat SG upon OVX, suggesting a pathologic involvement of ferroptosis in postmenopausal xerostomia.^[^ ^9^ ^]^

Ferroptosis, an iron‐dependent regulated cell death triggered by lipid peroxidation, is implicated in the progression of various diseases accompanied by redox imbalance and oxidative stress.^[^ ^10^ ^]^ Interestingly, the iron level is dynamically controlled during the menstrual cycle and upregulated after menopause,^[^ ^11^ ^]^ which may exacerbate postmenopausal osteoporosis and atherosclerosis.^[^ ^12^, ^13^ ^]^ In addition, a subset of ferroptosis‐associated genes has been proposed as a potential biomarker for the diagnosis of postmenopausal osteoporosis.^[^ ^14^ ^]^ Emerging evidence also indicates that ferroptosis affects the susceptibility to certain diseases, which varies depending on menopausal status or gender, with estrogen serving as a protective factor. Following the loss of nuclear factor erythroid 2‐related factor 2 (Nrf2) signaling induced by OVX, iron accumulates in the bone, leading to ferroptosis and subsequent osteoclastogenesis.^[^ ^15^ ^]^ In the murine acute kidney injury (AKI) model, female renal tubular epithelial cells exhibit higher resistance to ferroptotic damage compared to those from males, while either Nrf2 deletion or OVX diminishes the resilience of females to AKI.^[^ ^16^ ^]^ OVX also disturbs lipid metabolism in ApoE knockout mice by suppressing glutathione peroxidase 4 (GPX4) levels in endothelial cells (EC), resulting in ferroptotic EC loss and severe atherosclerosis.^[^ ^17^ ^]^ Moreover, estrogen receptor (ER) correlates with cancer cell susceptibility to ferroptosis, given that administration of an ER antagonist to ER^+^ cancer cells triggers ferroptotic death and subsequent tumor regression.^[^ ^18^, ^19^ ^]^ Inspired by these findings, we administered anti‐ferroptotic agents to OVX rats and reported their beneficial impact on the restoration of acinar cell markers in the context of estrogen deficiency.^[^ ^20^ ^]^ This work suggests a potential link between estrogen deficiency and iron accumulation/ferroptosis in the progression of xerostomia. However, the detailed understanding of how menopause influences SG cell components and their microenvironment, as well as the underlying molecular mechanisms that trigger menopause‐induced ferroptosis and SG dysfunction, remains largely unexplored.

To address these questions, here we investigated the transcriptomic changes underlying ferroptosis in the murine SG following OVX and conducted mechanistic studies using advanced SG‐derived organoids (SGOs). In particular, we focused on the TGFβ pathway with enhanced TGFβ2‐ TβRIII interaction as a potential key mediator in OVX‐induced SG dysfunction and explored its regulatory roles in the progression of ferroptotic damage within the SG. Finally, we verified the impacts of ferroptosis inhibitors on the functional recovery of SG in OVX mice, assessing their therapeutic benefits in the management of xerostomia.

## 2 Results

### 2.1 OVX Alters SG Structure and Reduces Saliva Secretion in Mice

**Figure 1:**
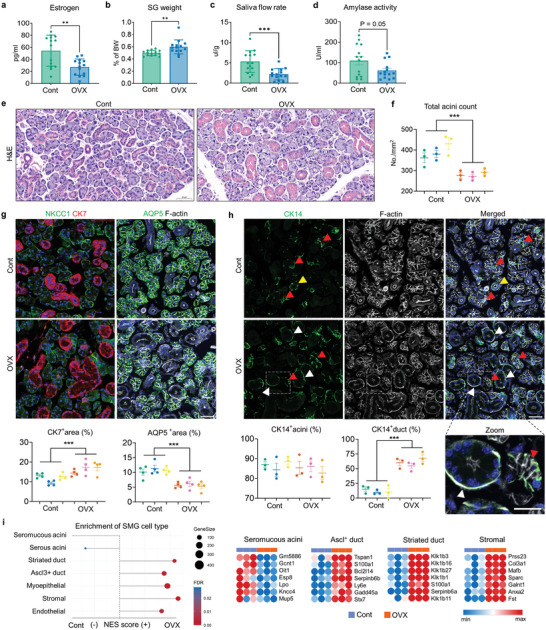
Comparison of SG structure between Cont‐ and OVX mice. Two months after OVX or sham surgery, a) serum estrogen levels, b) the ratio of SG weight to total body weight (BW), c) relative saliva secretion capacity and d) amylase activity in the secreted saliva were assessed in cont‐ and OVX mice (*n* = 14 for each group). e) Representative SG images of H&E staining and f) quantification results of total acini number. g) Immunofluorescence SG images showing acinar structure (NKCC1 and AQP5; green) and duct (CK7; red), with the area fraction occupied either by CK7 or AQP5 quantified. h) Immunofluorescence SG images showing the distribution of CK14^+^ (green) cells, with quantification results showing the proportion of CK14‐expressing acini and duct within the SG. Red arrowheads indicate CK14^+^ myoepithelial cells; yellow arrowheads and white arrowheads indicate CK14^+^ basal duct cells and ID cells, respectively. i) A dot plot depicting the gene set enrichment pattern for adult SMG cell types in the SG of Cont‐ and OVX mice and the Top 7 Gene set members on the rank‐ordered list. A total of three mice from each group were used for histological and microarray analysis. In g,h), F‐actin staining was performed to indicate the epithelial structure of the SG. Scale bar = 50 µm (e) and 40 µm (g, h). Data are shown as the mean ± SEM and compared by unpaired t‐test. **P < 0.01, ***P < 0.001.

8 weeks after the surgery, OVX mice exhibited a decline in serum estrogen levels and an increase in SG weight compared to the control group (**Figure** **1**a,b). Of note, OVX led to a reduction in the salivary flow rate in response to pilocarpine injection (Figure 1c). A trend toward lower amylase activity in the saliva of OVX mice than that of controls was also observed (Figure 1d), indicating that OVX group exhibited xerostomia‐like symptoms. Histological examination of SG tissues using H&E revealed a significant increase in the ductal compartment along with a corresponding reduction in the acinar‐occupied region in the OVX group (Figure 1e). The number of total acini was also significantly decreased due to estrogen deficiency (Figure 1f). Immunofluorescent staining with ductal cell marker cytokeratin 7 (CK7) or acinar markers including NKCC1 and aquaporin 5 (AQP5) verified the structural alterations of OVX‐SG (Figure 1g). Transcription levels of representative genes for duct (Ck7 and Ck19) and acini (*Aqp5, Prol1, Chrm1* and *Chrm3*) were altered in a manner consistent with histological analysis and immunostaining results, as determined by qPCR (Figure S1a, Supporting Information). Given that epithelial cytokeratin 14 (CK14) expression is dynamically regulated upon SG injury,^[^ ^21^ ^]^ we investigated the localization of CK14^+^ cells in the SGs. F‐actin was simultaneously stained with CK14 to visualize the acini and duct structures in the SG (Figure S1b, Supporting Information). In the homeostatic SG, CK14 signals were predominantly localized in the myoepithelial cells surrounding the acini (Figure 1h, red arrow) and few of basal duct cells were marked with CK14 (Figure 1h, yellow arrow). In contrast, an ectopic increase in the proportion of CK14‐labeled intercalated ducts (IDs) was observed following OVX (Figure 1h, white arrow), implying that OVX‐SG undergoes injury‐associated ductal regeneration. Next, we conducted a transcriptomic profiling array of SGs from control‐ and OVX mice and proceeded gene set enrichment analysis (GSEA) (Figure S2a, Supporting Information). Through a comparison of our data with gene signatures of adult SG cells (Table S1, Supporting Information),^[^ ^22^ ^]^ we observed a marked increase in duct‐associated gene expression concomitant with the downregulation of acinar cell genes in the OVX‐SG (Figure 1i; Table S2, Supporting Information). These findings demonstrate that OVX results in both a loss of acinar cells and an abnormal increase in the ductal compartment in the SG, leading to SG dysfunction.

### 2.2 TGFβ Signaling Activation and Redox Imbalance Accompanied by Ferroptosis are Observed in OVX‐SG

**Figure 2:**
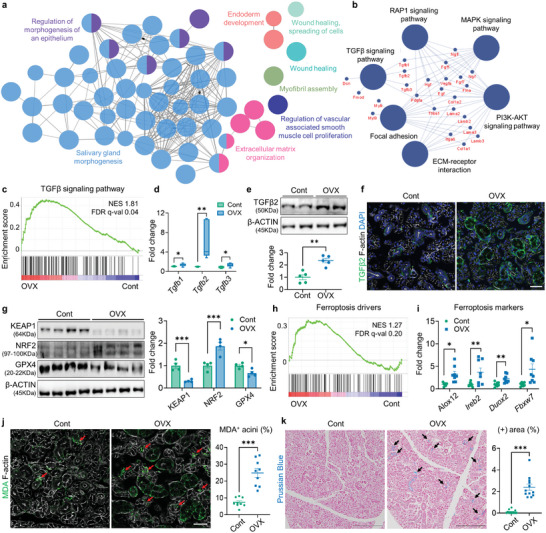
Transcriptome profiling reveals enrichment of TGFβ signaling and ferroptosis pathways in OVX‐SG. a,b) Visualization of functionally grouped networks via ClueGO, showing enriched GO‐BP terms (a) and KEGG pathways (b) within the Top three upregulated modules of OVX‐SGs. Hub genes shared in KEGG pathways are indicated. c) GSEA of Cont‐ and OVX‐SGs using a TGFβ signaling pathway gene signature extracted from Wiki Pathway. d) Comparison of *Tgfb1‐3* mRNA levels between Cont‐ and OVX‐SGs, conducted by qPCR. e) Analysis of TGFβ2 protein levels in Cont‐ and OVX‐SGs by Western blot (WB). f) Representative immunofluorescence images depicting TGFβ2 expression in the SG. g) Assessment of redox‐regulating protein levels in the SGs by WB. h) GSEA illustrating enrichment of ferroptosis drivers between Cont‐ and OVX‐SGs. i) Evaluation of relative mRNA expression levels of ferroptosis marker genes using qPCR. j) Detection and quantification of MDA^+^ acini (red arrows) relative to total acini in the SGs. k) Representative images of Prussian blue staining showing iron deposits (black arrows) within the SGs and quantification of the stained area. A total of three mice from each group were used for histological analysis. The number of biological replicates for WB and qPCR analysis corresponds to the number of dots on the graph. In (f) and (j), F‐actin staining was performed to indicate the epithelial structure of the SG. Scale bars = 40 µm (f, j) and 1 mm (k). Data are shown as the mean ± SEM and compared by unpaired t‐test. *P < 0.05, **P < 0.01, ***P < 0.001.

Using transcriptome profiling data, we explored the enriched pathways in control‐ and OVX‐SGs to identify the potential mechanisms underlying SG dysfunction upon estrogen deficiency. GSEA revealed that 31 of 50 terms among the Hallmark gene sets showed differential enrichment patterns in control or OVX samples (Figure S2b and Table S2, Supporting Information), indicating that OVX significantly alters the biological phenotype of the SG. A total of 823 differentially expressed genes (DEGs) of which 561 and 262 genes were up‐ and downregulated in the OVX‐SG, respectively (Table S3, Supporting Information). To estimate the molecular correlations between DEGs, Search Tool for the Retrieval of Interacting Genes (STRING)‐based Protein–Protein Interaction (PPI) networks were generated with increased or decreased DEGs then modules were identified by the Multi‐Contrast Delayed Enhancement (MCODE) algorithm. Subsequently, we selected the top three clusters in the upregulated network (Figure S2c and Table S4, Supporting Information) to study their functional enrichment pattern using ClueGO. The biological process among the Gene Ontology (GO‐BP) terms highly enriched for these modules were “SG morphogenesis”, “regulation of morphogenesis of an epithelium” and “extracellular matrix (ECM) organization” (**Figure** **2**a;Table S5, Supporting Information), in line with the observed structural changes of SG upon OVX (Figure 1). ClueGO KEGG analysis further indicated that upregulated top three modules in OVX‐SG were mainly involved in signal transduction pathways, including MAPK, TGFβ signaling and ECM receptor interaction (Figure 2b; Table S5, Supporting Information). Interestingly, all of the Tgfb isoforms, Tgfb1, 2 and 3, were identified as hub genes shared between multiple functional networks in the top three upregulated modules (Figure 2b; Figure S2d, Supporting Information) and TGFβ signaling‐related gene sets were significantly enriched in OVX‐SG (Figure 2c; Figure S2e, Supporting Information). Since Tgfb2 is the most prominently increased isoform at the transcriptional level after OVX (Figure 2d), we conducted further assessments of its protein level and found that OVX increased the abundance of TGFβ2 in the SGs (Figure 2e,f; Figure S3a, Supporting Information).

We have reported the redox imbalance accompanied by increased lipid peroxidation in the rat SG upon OVX.^[^ ^9^ ^]^ To determine whether similar phenomena occur in OVX mice, we assessed the activity of the ROS defense system, the KEAP1‐NRF2‐GPX4 axis, using western blotting analysis. The quantification results, as depicted in Figure 2g, indicate that OVX led to significant degradation of KEAP1, accumulation of NRF2 and a reduction in GPX4 in the SG, indicating an increment of ROS following estrogen deficiency. Similarly, gene sets for ferroptosis drivers and several lipid metabolism pathways were highly enriched in the OVX‐SG (Figure 2h; Figure S3b, Supporting Information) with increased mRNA transcripts for ferroptotic markers including *Fbxw7, Alox12, Ireb2* and *Duox2* (Figure 2i). Indeed, immunofluorescence staining for malondialdehyde (MDA), a ferroptosis indicator as an end‐product of lipid peroxides, revealed a considerable increase in the proportion of MDA‐labeled acini after OVX (Figure 2j; Figure S3c, Supporting Information). Given the importance of iron metabolism on ferroptosis regulation, we also estimated the intracellular iron pool by Prussian blue staining and found that OVX caused a massive iron deposition in the SG compared to controls (Figure 2k). These data collectively suggest a correlation between menopause and ferroptosis induction in the SG, partially attributable to aberrant iron homeostasis as well as a defect in the redox regulating system.

### 2.3 Mesenchymal Cells Isolated from OVX Mice Secrete TGFβ2, which Mediates SGO Damage

**Figure 3:**
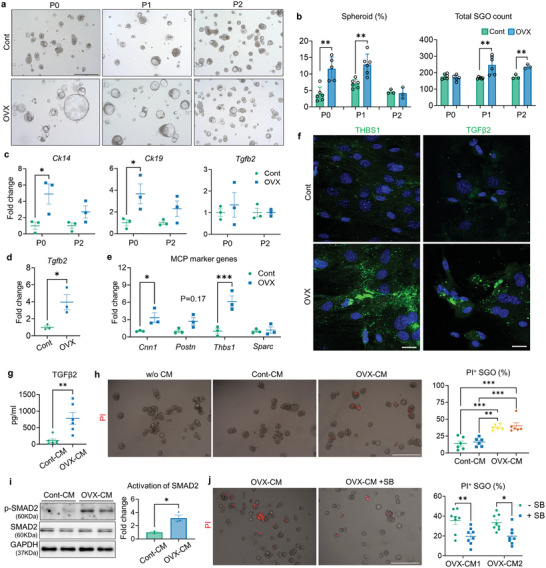
Characteristics of SGOs and SGMCs derived from Cont‐ and OVX mice. a) Representative images of SGOs derived from Cont‐ and OVX mice at day 7. b) Total count and proportion of spheroid‐shaped organoids assessed at day 7. c) qPCR results showing the mRNA expression levels of Ck14, Ck19 and *Tgfb2* in cont‐ and OVX‐SGOs at P0 and P2. d,e) qPCR results comparing the mRNA expression of *Tgfb2* (d) and MCP genes (e) between SGMCs isolated from Cont‐ and OVX mice. f) Immunofluorescence detection of THBS1 and TGFβ2 expression in SGMCs. g) ELISA‐based measurement of TGFβ2 levels released into the SGMC medium. h,i) SGOs were cultured with CMs from Cont‐ and OVX‐SGMCs (Cont‐CM and OVX‐CM) (*n* = 2 for each) for 48 h, then the impact of SGMC‐CMs on SGO viability (h) and Smad2 activation (p‐SMAD2/SMAD2 ratio) (i) were determined by PI staining and WB, respectively. j) SGOs were cultured with SGMC‐CM derived from OVX mice with or without SB treatment (10 µm) for 48 h, then their viability was assessed by quantification of PI^+^ SGOs. At least three lines of SGOs, SGMCs, and their CMs were used for all experiments, except for (h) and (j), where CMs were collected from two lines of SGMCs. Scale bar = 500 µm (a, h, i) and 40 µm (f). Data are shown as the mean ± SEM and compared by One‐way ANOVA with Dunnett`s multiple comparisons tests (h) or unpaired t‐test (rest of the analysis). *P < 0.05, **P < 0.01, ***P < 0.001.

Next, we conducted in vitro mechanical studies for OVX‐mediated SG damage using SGOs. In our floating culture system, SGOs expanded consistently over the passages (Figure S4a,b, Supporting Information). Cells comprising the SGOs were positive for epithelial lineage markers such as CD49f, EpCAM, CD24, and CD29 (Figure S4c, Supporting Information). We also confirmed the frequency of c‐kit^+^ progenitor‐like cells and EdU‐labeled cells was much higher in SGOs than that of conventionally‐cultured salisphere^[^ ^23^ ^]^ (Figure S4d, Supporting Information). These data indicate that SGOs contain a highly homogenous, proliferative population of the SG epithelium including SG stem cells (SGSCs). We then cultured SGOs from control‐ and OVX mice using our defined condition. SGOs derived from OVX mice (OVX‐SGOs) frequently exhibited a spheroid‐like morphology at the early passages (**Figure** **3**a,b) and their total count was greater than that of controls (Cont‐SGOs) (Figure 3b). The mRNA levels of duct markers, CK14 and CK19, were also elevated in OVX‐SGOs compared with the controls (Figure 3c), consistent with in vivo observation (Figure 1). It is noteworthy that, however, these differences disappeared with successive subcultures and neither *Tgfb2* (Figure 3c) nor ferroptosis marker *Fbxw7* (data not shown) was upregulated in OVX‐SGOs in transcriptional levels.

According to microarray‐based GSEA, the transcriptomic profiles of OVX‐SG showed positive correlations with the microenvironment‐associated gene sets as follows: SG stromal cells (Figure 1i); ECM organization (Figure 2a); ECM‐receptor interaction (Figure 2b); and collagen containing ECM (Figure S5a, Supporting Information). Therefore, we sought to determine how OVX affects the microenvironment of SG epithelium, by culturing SG mesenchyme‐derived stromal cells (SGMCs) (Figure S5b,c, Supporting Information). Notably, the relative amount of Tgfb2 transcripts in OVX‐SGMCs was higher than in Cont‐SGMCs (Figure 3d). We also screened the transcripts encoding non‐structural ECM components, so‐called matricellular protein (MCP), and noticed that the transcription of Thrombospondin 1 (*Thbs1*) and Calponin 1 (*Cnn1*) was significantly induced in OVX‐SGMCs (Figure 3e). Immunofluorescence analysis validated an abundant accumulation of THBS1 and TGFβ2 in the cytoplasm of OVX‐SGMCs (Figure 3f). Moreover, OVX‐SGMCs secreted significantly higher amounts of TGFβ2 into the medium compared to their counterparts (Figure 3g). Thus, SGMCs might play predominant roles in abnormal TGFβ2 production and ECM remodeling in the SG upon OVX.

**Figure 4:**
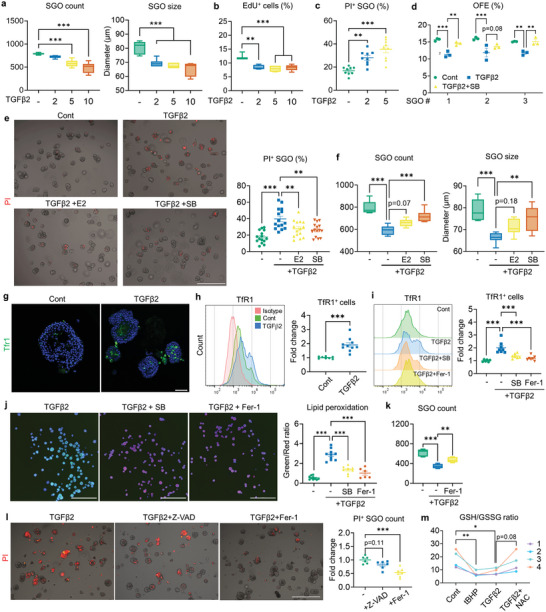
TGFβ2 impairs the growth and viability of SGOs by inducing ferroptosis. a–c) TGFβ2 treatment, initiated on day 4 and analyzed at day 7, resulted in a dose‐dependent decrease in the total count and size of SGOs (a), reduced the EdU incorporation ratio (b), and increased the number of PI^+^ SGOs (c). d) SB (10 µm) counteracts the negative impact of TGFβ2 (5 ng mL^−1^) on the OFE of SGOs. e,f) Co‐treatment with E2 or SB mitigated the TGFβ2 impact on SGOs, reducing cell death (e) and increasing the total organoid count and size in TGFβ2‐treated SGOs (f). g,h) Expression patterns of TfR1 in Cont‐ and TGFβ2‐treated SGOs were determined by immunostaining (g) and flow cytometry (h). i,j) Baseline ferroptosis in SGOs was estimated by TFR1 detection via flow cytometry (i) and lipid peroxidation assay (j). Induction of ferroptosis in TGFβ2‐treated organoids was impeded by SB and Fer‐1 (1 nM). k) Recovery of TGFβ2‐mediated reduction in SGO count with Fer‐1 co‐treatment. l) Viability of TGFβ2‐treated SGOs exposed to either Z‐VAD (10 µm) or Fer‐1 assessed by PI staining. Quantification analysis highlights the dominance of ferroptosis over apoptosis in TGFβ2‐induced SGO damage. m) Reduced‐ and oxidized GSH levels in SGOs were evaluated after 24 h of TGFβ2 treatment. tBHP was used to induce oxidative stress, while antioxidant NAC was co‐treated with TGFβ2 to recover GSH depletion. At least three lines of SGOs were used for all experiments. For (g) and (i), Hoechst staining was conducted to visualize each organoid for analysis. Scale bars = 500 µm (e, l), 40 µm (g) and 1 mm (j). Data are shown as the mean ± SEM and compared by one‐way ANOVA with Dunnett`s multiple comparisons test. *P < 0.05, **P < 0.01, ***P < 0.001.

To address the pathologic impact of OVX‐SGMCs on SG epithelial cells, we co‐cultured SGOs and SGMCs then evaluated SGO viability by PI labeling. Compared to their counterparts, SGOs cultured on OVX‐SGMCs demonstrated a higher frequency of PI^+^ organoids (Figure S5d, Supporting Information). Intriguingly, conditioned media (CM) could reproduce the detrimental impact of OVX‐SGMCs on SGO survival (Figure 3h), which suggests a contribution of secretory factor(s) to this phenomenon. We noticed that CM from OVX‐SGMCs (OVX‐CM) promoted the phosphorylation of SMAD2 in SGOs (Figure 3i), while co‐treatment with a potent inhibitor of the TGFβ pathway, SB431542 (SB), mitigated the cytotoxic impact of OVX‐CM (Figure 3j); accordingly, the activation of canonical TGFβ‐SMAD signaling pathway might underlie the SG epithelial damage upon OVX, with stromal TGFβ2 acting as a key mediator. Indeed, direct treatment of TGFβ2 led to a drastic reduction in SGO formation (**Figure** **4**a; Figure S6a, Supporting Information), as corroborated by quantitative analysis of EdU^+^ proliferating cells (Figure 4b; Figure S6b, Supporting Information) and PI^+^ dying organoids (Figure 4c), while the pre‐treatment of SB reversed the negative impact of TGFβ2 on SGO formation (Figure 4d). Based on previous works reporting that estrogen can intervene TGFβ signal transduction by regulating SMAD2/3 pathway,^[^ ^24^ ^]^ we co‐treated Estradiol (E2) with TGFβ2 to SGOs. Notably, SMAD2 phosphorylation induced by TGFβ2 treatment was suppressed by E2, as well as SB (Figure S6c, Supporting Information) and impaired cell viability and proliferative capacity of TGFβ2‐exposed SGOs were normalized following E2 treatment (Figure 4e; Figure S6d, Supporting Information), resulting in the recovery of organoid growth (Figure 4f). Therefore, we concluded that SMAD activation functions as a downstream regulator of TGFβ2‐driven SGO damage.

### 2.4 TGFβ2 Treatment Leads to Ferroptosis in SGOs

We noticed that the administration of TGFβ2 induced a significant increase in the transcriptional activity of ferroptosis‐associated genes such as *Duox1*, *Alox12*, and *Fbxw7* in SGOs (Figure S7a, Supporting Information) along with an upregulation of ferroptosis indicator transferrin receptor 1 (TfR1), a major iron‐specific importer (Figure 4g,h). We monitored the lipid peroxidation change in SGOs using a fluorescence lipid sensor that undergoes color change from red to green upon peroxidation (Figure S7b, Supporting Information) and found that TGFβ2‐treated SGOs exhibited an enhanced lipid peroxidation compared to controls, which could be prevented by SB treatment (Figure 4i; Figure S7c, Supporting Information). Of note, the administration of a ferroptosis inhibitor, Ferrostatin‐1 (Fer‐1), not only suppressed TGFβ2‐mediated lipid peroxidation and TfR1 upregulation in SGOs (Figure 4i,j) but also rescued them from retarded growth and cell death (Figure 4k,l), while pan‐caspase inhibition by Z‐VAD‐FMK (Z‐VAD) couldn`t curb the cytotoxic impact of TGFβ2 on SGOs (Figure 4l). We also found that TGFβ2 treatment resulted in the depletion of the reduced glutathione (GSH) pool in SGOs (Figure 4m), suggesting its correlation to redox imbalance. Accordingly, an iron chelator DFO and antioxidant NAC exhibited a similar protective impact against TGFβ2‐mediated toxicity (Figure S7d, Supporting Information). These results collectively suggest that iron‐dependent ferroptosis, rather than apoptosis, functions as a major pathologic mechanism underlying TGFβ2‐induced epithelial injury in the SG.

### 2.5 Ferritin Degradation Process via Ferritinophagy is Accelerated by TGFβ2 Treatment

**Figure 5:**
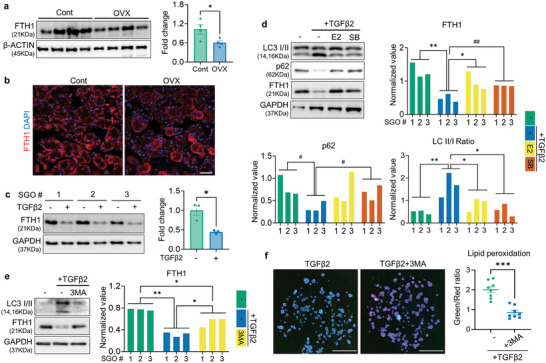
TGFβ2 induces ferritinophagy in SGOs. a,b) FTH1 expression in SG tissues of Cont‐ or OVX mice was analyzed by WB (a) and immunofluorescence (b). c,d) SGOs were treated with TGFβ2, SB, and E2 as indicated and analyzed by WB. TGFβ2 treatment led to a downregulation of FTH1 protein levels in SGOs (c), while co‐treatment with E2 or SB restored autophagic flux and FTH1 levels in TGFβ2‐exposed SGOs (d). e,f) 3MA (2.5 mm) was co‐treated with TGFβ2 to SGOs to intervene TGFβ2‐meidated ferritinophagy. The basal levels of autophagic flux and FTH1 protein (e) and lipid peroxidation in TGFβ2‐treated SGOs (f) were normalized by the autophagy inhibitor 3‐MA. At least 3 lines of SGOs were used for all experiments. Hoechst staining was conducted in (f) to visualize each organoid for the analysis. Scale bar = 40 µm (b) and 1 mm (f). Data are shown as the mean ± SEM and compared by unpaired t‐test (a, c, f) or one‐way ANOVA with Dunnett`s multiple comparisons tests (d, e). *P < 0.05, **P < 0.01, ***P < 0.001. For (d), ^#^ *P* < 0.05 and ^##^ *P* < 0.01, where the statistical significance was determined by unpaired t‐test.

To gain mechanistic insight into how TGFβ2 triggers ferroptosis in the SG epithelium, we investigated the expression profiles of key modulators involved in lipid peroxidation‐associated redox signaling in control‐ and OVX‐SG. We found a remarkable reduction in iron storage protein, ferritin heavy chain 1 (FTH1), in OVX‐SG (**Figure** **5**a,b). Notably, both TGFβ2 and CM derived from OVX‐SGMCs exhibited similar suppressive effects on FTH1 expression in SGOs (Figure 5c; Figure S8a, Supporting Information). Considering that the mRNA level of Fth1 in TGFβ2‐treated SGOs was comparable to that of control (Figure S8b, Supporting Information), TGFβ2 might regulate the FTH1 expression at the protein level. The degradation process of ferritin primarily resides in autophagy‐mediated turnover so‐called ferritinophagy.^[^ ^25^ ^]^ Therefore, we evaluated the differences in basal autophagic activity between Cont‐ and OVX‐SGs by analyzing autophagosomal markers using WB and found that the LC3‐II level in the SG was significantly upregulated upon OVX (Figure S8c, Supporting Information). In addition, administration of TGFβ2 increased LC3‐II/I ratio accompanied by the downregulation of p62 in SGOs and blocking of autophagosome‐lysosome fusion with bafilomycin A1 (BafA1) accumulated both LC3‐II and p62 in TGFβ2‐treated SGOs (Figure 5d; Figure S8d, Supporting Information). Moreover, TGFβ2‐mediated autophagic flux was dramatically suppressed by either SB or E2 treatment as evidenced by the reversal of LC3 I/II and p62 levels, in parallel to a restoration of FTH1 expression (Figure 5d). These findings led us to ask whether TGFβ2 can provoke the ferritinophagy process. Accordingly, we blocked the autophagy initiation by treating 3‐methyladenine (3MA), an inhibitor of autophagosome formation. Interestingly, 3MA prevented the loss of FTH1 (Figure 5e) and significantly reduced the extent of lipid peroxidation in the TGFβ2‐treated group (Figure 5f). Therefore, degradation of FTH1 via ferritinophagy is required for TGFβ2‐induced ferroptotic damage in SG epithelial cells.

### 2.6 Knockdown of type III TGFβ Co‐Receptor (TβRIII) Hinders the Pathologic Impact of TGFβ2 on SGOs

**Figure 6:**
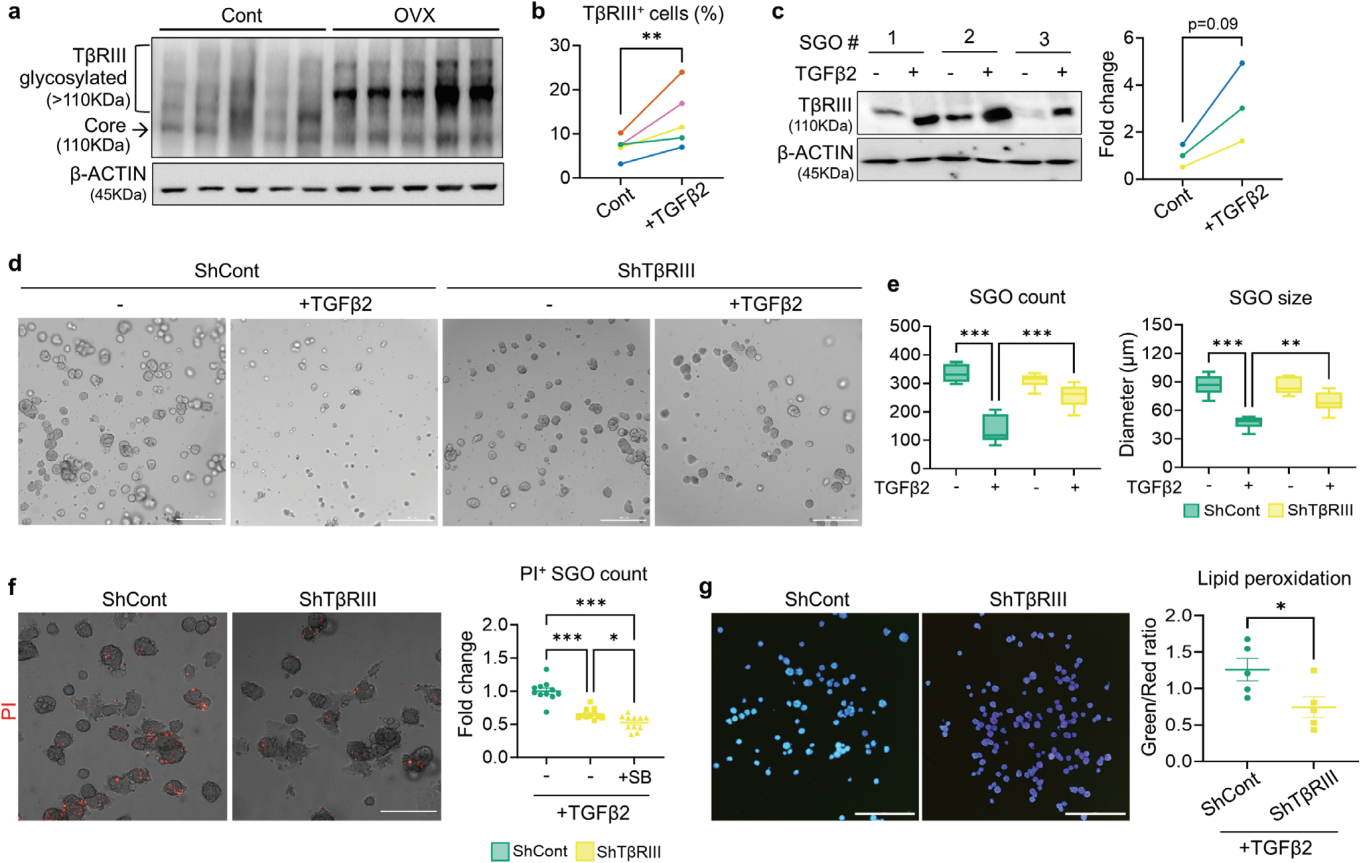
TβRIII is involved in a detrimental action of TGFβ2 on SGOs. a) Representative WB image detecting TβRIII in the SG of cont‐ and OVX mice (*n* = 5 for each). b,c) The impact of TGFβ2 on TβRIII expression in SGOs was investigated with flow cytometry (b) and WB (c). d–f) Representative images of shCont‐ and shTβRIII‐SGOs after TGFβ2 exposure (d) and their growth parameters (e) as well as viability assessment (f). g) Lipid peroxidation levels in shCont‐ and shTβRIII‐SGOs were evaluated after TGFβ2 treatment. TGFβ2 was administered on culture day 5 for 72 h (c‐e) or 24 h (f, g). At least three lines of SGOs were used for all experiments. Hoechst staining was conducted in (g) to visualize each organoid for the analysis. Scale bar = 300 µm (d, f) and 1 mm (g). Data are shown as the mean ± SEM and compared by unpaired t‐test (b, c, g) or one‐way ANOVA with Dunnett`s multiple comparisons tests (e, f). *P < 0.05, **P < 0.01, ***P < 0.001.

Since TGFβ2 demonstrates lower affinity for designated signaling receptors type I and II compared to TGFβ1 and TGFβ3, it relies on the TβRIII to facilitate its binding to the receptor complex, thus ensuring effective downstream signal transduction.^[^ ^26^ ^]^ Interestingly, both core and modified forms of TβRIII were significantly increased in the OVX‐SGs (**Figure** **6**a). Moreover, treatment of TGFβ2 directly upregulated TβRIII expression in SGOs, as evidenced by flow cytometry (Figure 6b) and WB analysis (Figure 6c). Hence, we assumed that TβRIII is involved in the detrimental action of TGFβ2 on SG. To test this hypothesis, we generated lentivirus harboring TβRIII‐targeted shRNA and transfected it into SGOs to silence TβRIII expression. We verified the knockdown efficiency of TβRIII‐targeted shRNA in both normal‐ (Figure S8e, Supporting Information) and TGFβ2‐stimulated SGOs (Figure S8f, Supporting Information). No significant difference was observed in the growth pattern of shCont‐ and shTβRIII‐transfected SGOs in homeostatic condition; meanwhile, even in the presence of TGFβ2, the growth of shTβRIII‐bearing SGOs was not compromised compared to counterpart (Figure 6d,e). Indeed, silencing of TβRIII significantly protected SGOs against the toxicity mediated by TGFβ2, as corroborated by the reduced proportion of PI‐labeled organoids (Figure 6f). Moreover, transfection of shTβRIII in SGOs mitigated the induction of lipid peroxidation following treatment of TGFβ2 (Figure 6g) or CM derived from OVX‐SGMCs (Figure S8g, Supporting Information). These results indicate that TβRIII plays a pivotal role in mediating TGFβ2‐induced SG dysfunction, with its expression notably increased following OVX.

### 2.7 Long‐Term Administration of Ferroptosis Inhibitor Liproxstatin‐1 Protects Against SG Dysfunction in OVX Mice

**Figure 7:**
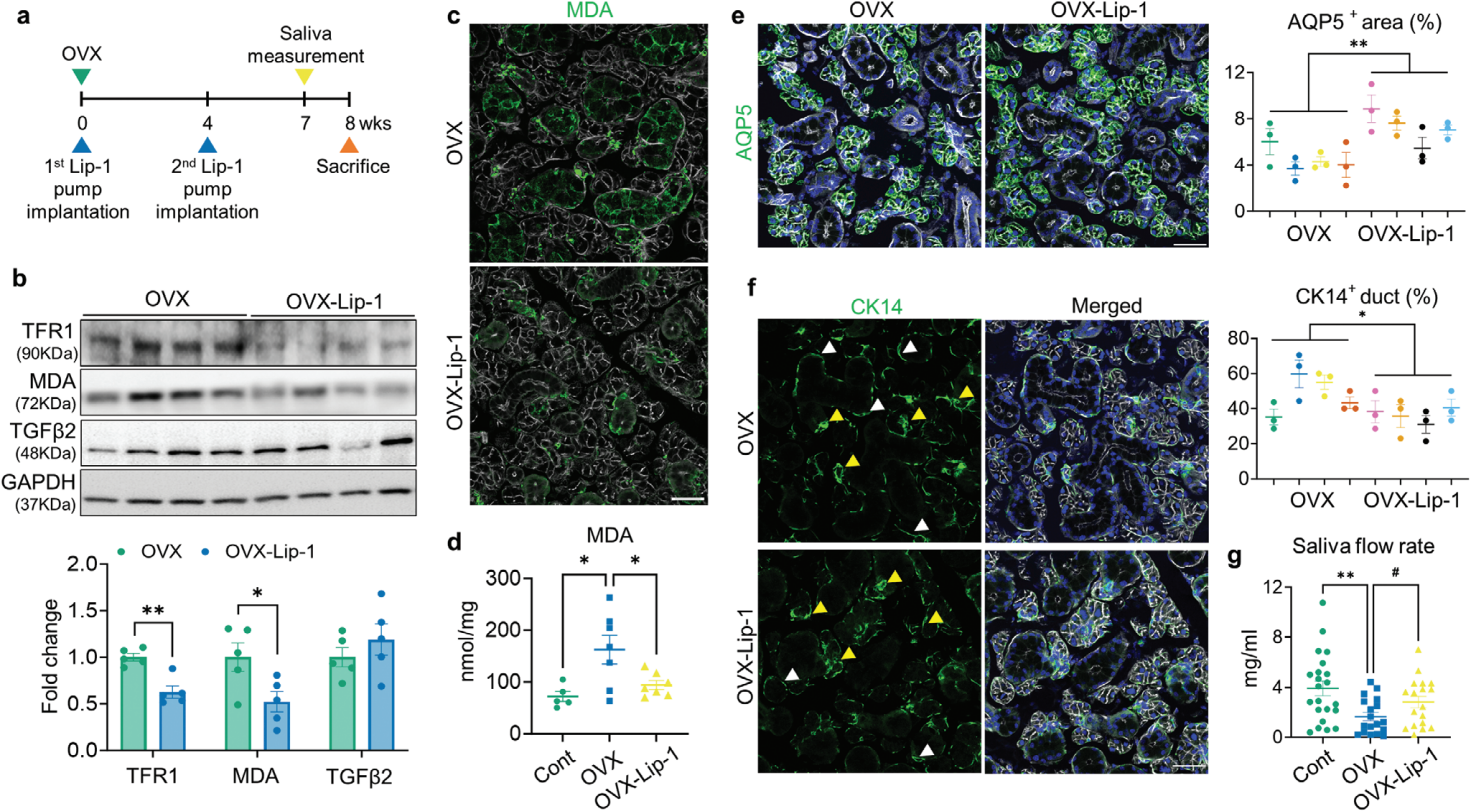
In vivo administration of ferroptosis inhibitor contributes to SG recovery in OVX mice. a) Experimental timeline schematic for the animal study. b) Immunoblot analysis showing the influence of Lip‐1 treatment on the regulation of ferroptosis, lipid peroxidation and TGFβ2 level in the OVX‐SGs. c,d) Representative images of MDA immunostaining (c) and quantification of MDA levels (d) within the SG tissues, demonstrating the beneficial impact of Lip‐1 treatment in reducing lipid peroxidation levels in OVX‐SGs. e) The quantity of acini in the SG was estimated by AQP5 immunostaining. f) SG images labeled with CK14 and assessment of the proportion of CK14^+^ ducts. CK14‐expressing acini and duct are indicated by yellow and white arrowheads, respectively. g) 7 weeks after OVX or sham surgery, the saliva secretory capacity of each group upon pilocarpine injection was measured. A total 4 mice for each group were used for histological analysis. The number of biological replicates for the WB analysis, MDA measurement and saliva measurement corresponds to the number of dots on the graph. In (c, e, f), F‐actin staining was performed to indicate the epithelial structure of the SG. Scale bar = 40 µm. Data are shown as the mean ± SEM and compared by unpaired t‐test (e, f) or one‐way ANOVA with Dunnett's multiple comparisons tests (d, g). *P < 0.05, **P < 0.01. For (g), ^#^ *P* < 0.05, where the statistical significance was determined by unpaired t‐test.

To determine whether the suppression of ferroptosis contributes to the alleviation of SG dysfunction in OVX mice, we implanted an osmotic pump containing Liproxstatin‐1 (Lip‐1), a potent ferroptosis inhibitor, in OVX mice (**Figure** **7**a). Although Lip‐1 treatment did not mitigate the elevated TGFβ2 expression, it effectively reduced the protein levels of TfR1 and MDA (Figure 7b) and attenuated the aberrant deposition of MDA in OVX‐SG (Figure 7c); indeed, OVX resulted in more than a two‐fold increase in MDA levels compared to controls, whereas Lip‐1 treatment reversed this increase, restoring MDA production to those similar to control mice (Figure 7d). Furthermore, immunofluorescent staining for AQP5 and CK14 revealed that Lip‐1 not only increased the proportion of the acini compartment (Figure 7e; Figure S9a, Supporting Information) but also suppressed the emergence of CK14‐positive ducts (Figure 7f; Figure S9b, Supporting Information) in OVX‐SGs, indicating that structural alterations in OVX‐SGs can be normalized by Lip‐1 infusion. Lip‐1 did not affect BW or relative SG weight in OVX mice (Figure S9c,d, Supporting Information). On the contrary, Lip‐1‐infused OVX mice exhibited enhanced saliva secretion compared to their counterparts (Figure 7g), with no significant difference in salivary amylase activity between the groups (Figure S9e, Supporting Information). These data demonstrate the therapeutic impact of ferroptosis inhibition on alleviating SG impairment in OVX mice.

### 2.8 TGFβ2 Induces SG Epithelial Damage in Humans

**Figure 8:**
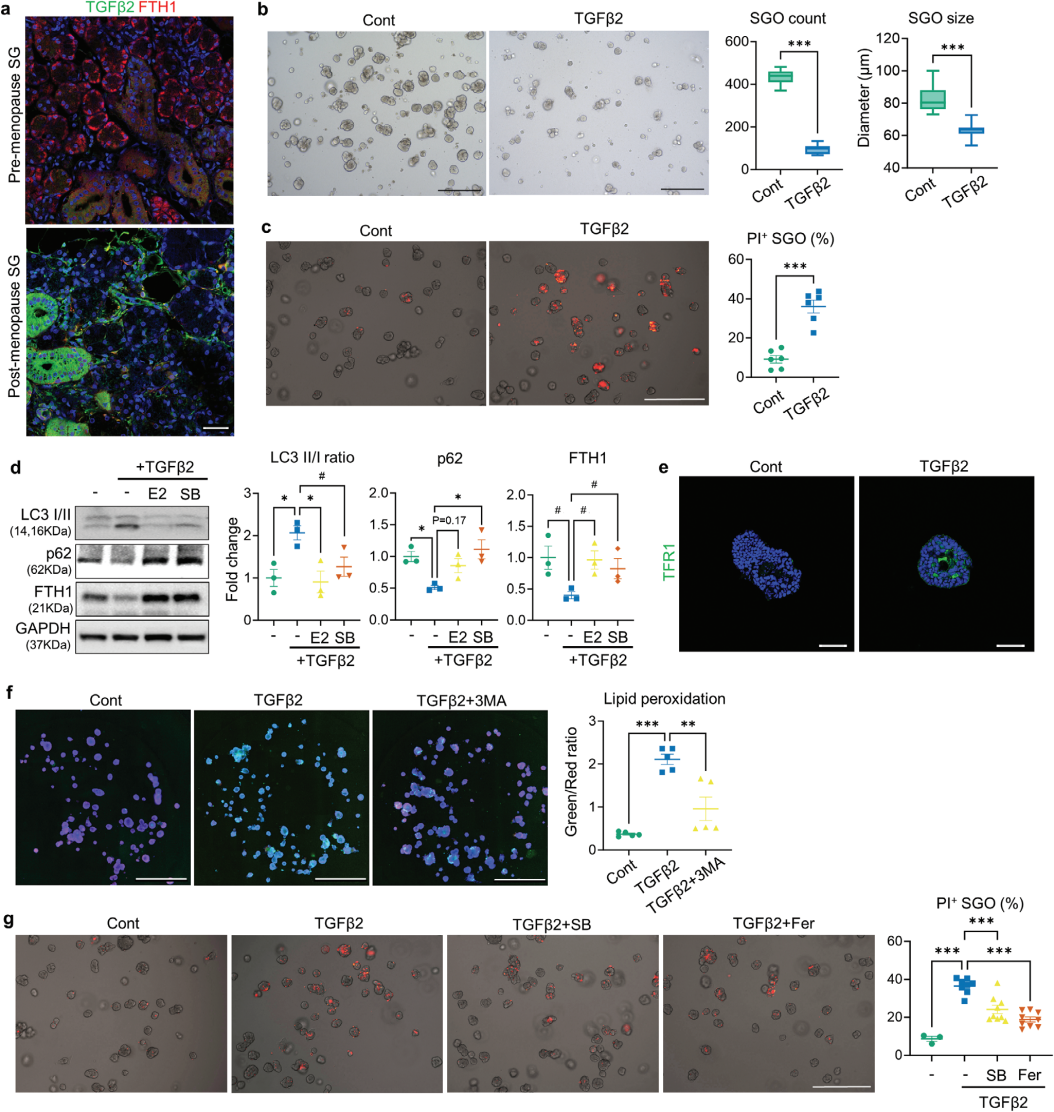
Validation of the detrimental impact of TGFβ2 on human SG epithelium using tissue samples and organoids (hSGOs). a) Representative immunofluorescence images showing TGFβ2 and FTH1 expression patterns in SG sections derived from pre‐ and postmenopausal women. b,c) TGFβ2 treatment was applied to hSGO cultures on day 4. Both the growth (b) and viability (c) of hSGOs were impaired by TGFβ2. d) Immunoblot analysis focusing on autophagy proteins and FTH1 was performed on hSGOs in the presence of indicated chemicals. e) Immunofluorescent images showing TFR1 expression in Cont‐ and TGFβ2‐treated hSGOs. f) 3MA treatment reduced the lipid peroxidation levels in TGFβ2‐treated hSGOs. g) Quantification of PI^+^ hSGOs indicating the protective effect of SB and Fer‐1 against TGFβ2‐mediated cell death. In (f), Hoechst staining was conducted to visualize each organoid for the analysis. Scale bar = 40 µm (a, e), 500 µm (b, c, g) and 1 mm (f). Data are shown as the mean ± SEM and compared by unpaired t‐test (b, c) or one‐way ANOVA with Dunnett`s multiple comparisons test (d, f, g). **P < 0.01, ***P < 0.001.

Drawing on the present observations from our mouse models, we explored whether the pathological changes linked to TGFβ activation and ferroptosis are also evident in human SGs. Notably, immunohistochemical analysis demonstrated the pronounced expression of TGFβ2 in the SG, particularly surrounding the ductal cells and mesenchyme, of postmenopausal women (**Figure** **8**a; Figures S10 and S11, Supporting Information). Moreover, the distribution pattern of FTH1 showed significant differences between the two groups: In the SG of premenopausal women, FTH1 expression was evenly distributed in both acini and ducts; however, its expression in acini was markedly reduced in the SG of postmenopausal women, resulting in an overall reduction in FTH1 immunoreactivity (Figure 8a; Figures S10 and S11, Supporting Information). Next, we validated the pathologic implication of TGFβ2 in SG epithelial damage in humans with human‐derived SGOs (hSGOs). As observed in mouse SGOs, TGFβ2‐treated hSGOs exhibited a marked growth impairment with an increased percentage of PI^+^ organoids (Figure 8b,c). Dysregulation of FTH1 concomitant with enhanced autophagic flux was also evident in TGFβ2‐treated hSGOs (Figure 8d). Indeed, TGFβ2 upregulated the basal level of TfR1 expression (Figure 8e) and lipid peroxidation in hSGO, while this phenomenon was reversed by the addition of 3MA (Figure 8f). Finally, the suppression of SMAD signaling with SB or blocking of ferroptosis with Fer‐1 could protect hSGOs against TGFβ2‐mediated cell death (Figure 8g). Collectively, TGFβ/SMAD signaling induces ferritinophagy and subsequently leads to ferroptotic cell death in the human SG epithelium.

## 3 Discussion

Utilizing the mouse OVX model, here we demonstrated that estrogen deficiency induces structural changes and functional defects in the SGs. Through microarray‐based transcriptome profiling and functional enrichment analysis, we delineated the molecular landscape in the SG following OVX and observed an aberrant activation of ferroptosis and TGFβ signaling pathway with enhanced expression of TGFβ2. Notably, TGFβ2 treatment in SG epithelial cells stimulates ferritinophagy‐mediated degradation of ferritin, which triggers iron‐dependent lipid peroxidation and ferroptotic cell death. Furthermore, the administration of a potent ferroptosis inhibitor, Lip‐1, can restore saliva secretion in OVX mice, highlighting the detrimental role of ferroptosis in the progression of postmenopausal xerostomia.

Menopause and the subsequent decrease in estrogen level compromise tissue homeostasis, resulting in diverse pathological alterations.^[^ ^27^ ^]^ These changes include reduced acini secretory complex and ductal dilation in the SG,^[^ ^9^, ^28^ ^]^ which were evident in our mouse OVX model. We also found the upregulation of TGFβ and MCP expression in OVX‐SG, indicating active ECM remodeling. TGFβ signaling pathway plays a crucial role in the morphogenesis and homeostasis of SG; both overexpression or deletion of TGFβ hinders SG development.^[^ ^29^, ^30^ ^]^ Moreover, SG injury induces an upregulation of TGFβ expression to promote wound healing, although uncontrolled TGFβ signaling can result in pathological consequences. For instance, excessive TGFβ activation is observed in the SGs of patients with radiotherapy‐induced xerostomia^[^ ^31^ ^]^ or Sjögren's syndrome,^[^ ^32^ ^]^ while both duct ligation and exposure to irradiation induce ectopic TGFβ expression in the murine SG.^[^ ^33^, ^34^, ^35^ ^]^ Based on our data, OVX markedly increases TGFβ2 expression in the SG relative to other isoforms. This might stem from the localization patterns of each isoform within the SG. In mature human SG, TGFβ2 is predominantly expressed in the duct, while TGFβ1 and TGFβ3 are localized at the acinar cells and myoepithelial cells, respectively.^[^ ^36^ ^]^ Therefore, the histological changes in the OVX‐SG, characterized by a reduction in acini and an increase in duct compartments, might influence the differential expression of TGFβ isoforms. We also propose SGMCs as an additional source of TGFβ2 production in OVX‐SG. Given that OVX‐SGMCs compromise the survival of SGOs, estrogen deficiency might alter the mesenchymal phenotype, which in turn leads to epithelial cell damage. Although no clear signs of collagen deposition or inflammation were yet observed in the SG of OVX mice, this phenomenon potentially reflects the progression of SG fibrosis after menopause. Recent work by Sun et al. demonstrated that TGFβ2 is highly expressed in the lungs of idiopathic pulmonary fibrosis (IPF) patients owing to its lower activation threshold than that of TGFβ1, elucidating a distinct role of TGFβ2 in the progression of IPF.^[^ ^37^ ^]^ This aligns with our hypothesis that, under estrogen‐deficient conditions, TGFβ2 upregulation precedes that of TGFβ1 to mediate initial responses as the starting point for the fibrotic cascade in the SG. From this perspective, exploring the therapeutic potential of selective TGFβ inhibition in improving SG function could be an intriguing topic for future research.

Given the well‐documented antioxidant properties of estrogen,^[^ ^38^ ^]^ it wouldn`t be surprising that impaired redox signaling and ferroptosis mediate SG dysfunction upon menopause. Compelling evidence of active ferroptosis is found in the SG following OVX, including abnormal iron deposits, increased transcription of ferroptosis marker genes, and accumulation of reactive lipid peroxide MDA. OVX disrupts the homeostasis of the KEAP1/NRF2 pathway, a major antioxidant regulatory system. In specific, even with elevated NRF2 levels, the reduced expression of GPX4 in the OVX‐SG suggests that endogenous ROS is not being adequately controlled following estrogen deficiency, potentially resulting in ferroptosis. We found that supplement of E2 efficiently protects against ferroptotic cell damage in SGOs, providing indirect but substantial evidence for the pivotal role of estrogen in preventing ferroptosis in the female SG. It is worth noting that the activity of estrogen signaling determines the susceptibility to ferroptosis. For instance, sulfasalazine exerts a tumor‐suppressive role by inducing ferroptosis, primarily through the upregulation of the iron importer TFRC, and this effect is negatively correlated with estrogen receptor (ER) expression levels in cancer cells.^[^ ^39^ ^]^ Also, one of the novel transcriptional targets of ER, membrane‐bound O‐acyltransferase domain‐containing 2 (MBOAT2), suppresses ferroptosis by modulating the phospholipid peroxidation process.^[^ ^40^ ^]^ Our study supports these findings and underscores the need to elucidate how estrogen signaling modulates ferroptosis in the SG. Further investigation into identifying the specific cell types involved in ROS production and lipid metabolism will also contribute to the development of ferroptosis‐targeted therapies for menopausal xerostomia.

Meanwhile, we also demonstrate that a decline in ferritin level, primarily attributed to its enhanced degradation, serves as the key driver of excessive ferroptosis in the OVX‐SG. As an iron‐storage protein, ferritin is ubiquitously expressed and responsible for the oxidation of reactive ferrous iron (Fe2^+^) to non‐toxic ferric (Fe3^+^) form. Therefore, a deficiency of ferritin elevates the Fe2^+^ level in the cytoplasm, which triggers the Fenton reaction and ROS generation.^[^ ^10^ ^]^ According to the previous report, TGFβ1 can lead to a depletion of intracellular glutathione by downregulating xCT, an essential component of the cystine/glutamate antiporter, thereby inducing ferroptosis in hepatocellular carcinoma cells.^[^ ^41^ ^]^ Here we discovered that TGFβ2 induces lipid peroxidation and ferroptotic damage in SGOs, accompanied by a marked decrease in ferritin levels. These phenomena can be mitigated by an autophagy blocker, indicating that TGFβ2 promotes ferritin degradation via ferritinophagy. Thus, our findings demonstrate a novel mechanism, ferritinophagy, by which TGFβ signaling can intervene in ferroptosis. Notably, histological examination of pre‐ and postmenopausal SG reveals a downregulation of ferritin levels within the acini compared to the duct region. This result could be explained by either a reduction in the absolute number of acini due to their limited regenerative capacity following injury,^[^ ^21^, ^42^ ^]^ or by an acini‐specific decline in ferritin expression for unknown reasons. Concurrent with the predominant MDA expression in the acini of OVX‐SG, these findings implicate an enhanced vulnerability of acinar cells to ferroptotic damage. Further research is required to establish the causal relationship between acini loss and the reduction in acini ferritin levels during the development of menopausal xerostomia. Intriguingly, women exhibit elevated serum ferritin levels after menopause in general.^[^ ^43^ ^]^ While it is primarily attributed to the cessation of menstrual blood loss, inflammation and metabolic alteration following menopause can stimulate ferritin secretion mainly from macrophages,^[^ ^44^ ^]^ but also from damaged or ruptured cells.^[^ ^45^, ^46^ ^]^ Given that extracellular ferritin functions as an additional iron source to cells,^[^ ^47^, ^48^ ^]^ serum ferritin might exacerbate iron‐mediated damage in SG cells, where the ferritinophagy process is already activated. Thus, investigating the capacity of SG cells to uptake serum ferritin and identifying the receptor responsible for this process might unveil novel therapeutic targets for postmenopausal xerostomia.

Organoid is a self‐assembling 3D construct derived from stem cells, designed to replicate the structural and functional characteristics of tissue or organ. Here, we utilized SGOs to overcome the lack of appropriate SG‐derived cell lines for our research. Instead of the conventional Matrigel‐rich dome‐based method,^[^ ^49^, ^50^ ^]^ we developed a floating culture method that supports consistent propagation of SGOs with stable SGSC marker expression. Notably, TGFβ2 exerts similar cytotoxic effects on both murine‐ and human SGOs, suggesting the pathogenic mechanisms underlying TGFβ‐mediated ferroptosis are well conserved in the SG epithelium. Since the majority of in vitro experiments in this study were conducted with undifferentiated SGOs, the influence of TGFβ2 on mature SGOs needs to be investigated to elucidate the cell‐specific pathologic impact of TGFβ2 on acinar and ductal cells and to validate present in vivo findings. Therefore, further effort in finding chemical combinations as FBS substitutes is needed to refine the SGO differentiation protocol for the generation of acini or duct‐enriched SGOs.

## 4 Conclusion

In summary, we suggest that ferroptosis is implicated in SG damage following menopause, with TGFβ signaling identified as a key mediator, particularly in promoting ferritin degradation via ferritinophagy. Our study demonstrates that the application of ferroptosis inhibitor restores SG function in OVX mice, highlighting its therapeutic potential for managing xerostomia and improving the quality of life for postmenopausal women.

## 5 Experimental Section

### Animals and Experimental Design

After the stabilization period for 1 week, 6‐week‐old female BALB/c mice (Koatech, South Korea) were anesthetized using 2,2,2‐Tribromoethanol (avertin; 0.25 mg kg^−1^) (Sigma, St. Louis, MO) and either a bilateral OVX (designated as OVX group) or sham surgery (designated as Cont group) was performed as previously.^[^ ^9^ ^]^ To validate the efficacy of OVX, serum estrogen concentrations were compared between the OVX‐ and Cont groups using a competitive ELISA‐based estrogen quantification kit (Mybiosource, San Diego, CA). To suppress ferroptosis in vivo, anti‐ferroptotic agent Liproxstatin‐1 (Lip‐1; Cayman, Ann Arbor, MI) was administrated to the OVX group as described in the following section. 8 weeks later, the mice were euthanized and their SGs (submandibular gland; SMG) along with blood samples were collected for further analysis.

### Implantation of Osmotic Pump Containing Lip‐1 Solution

For the continuous and consistent delivery of Lip‐1 to OVX mice throughout the in vivo experiment (8 weeks), osmotic minipumps (model 1004; DURECT Corporation, Cupertino, CA) were prepared, filled with either a Lip‐1 solution (dissolved in a 1:1 ratio of DMSO to water; estimated delivery dose: 7.5 mg kg^−1^/day) or vehicle and implanted them intraperitoneally following the OVX. Given that the guaranteed delivery period of model 1004 was 28 days, the initial minipumps were replaced with new ones 4 weeks after the first implantation.

### Saliva Collection and Determination of Amylase Activity

7 weeks post‐surgery, the mice were anesthetized and administered an intraperitoneal injection of pilocarpine (5 mg kg^−1^, Sigma) to stimulate saliva secretion. After a 15‐min‐long saliva collection, the capacity for saliva production, which can represent the SG function, was calculated as the ratio of the total weight of collected saliva (mg) to the body weight (g) of each subject. The amylase activity of saliva was determined using an amylase assay kit (Abcam, Cambridge, MA), adhering to the manufacturer's protocol.

### Tissue Processing and Histopathologic Assessment

For histological evaluation of the SGs, paraffin‐embedded tissue blocks were sectioned into 5 µm‐thick slices and stained with hematoxylin and eosin (H&E) to visualize the overall structure of the SGs. The presence of iron deposits within the SGs was detected using Prussian Blue staining, which labels ionic iron as blue dots. The area of Prussian Blue‐positive signals within the whole tissue section was quantified using NIH Image J software (ver 1.53t). Each staining procedure was performed using specific stain kit purchased from Abcam.

### Microarray Data Acquisition, GSEA, PPI Network Construction and Module Analysis for DEGs

Microarray‐based transcriptomic profiling of SMGs (*n* = 3 for each group) was conducted 8 weeks after the OVX using the Clariom S Assay microarray platform (Thermo Fisher Scientific; Thermo, Waltham, MA). Total tissue RNA was extracted with the Trizol method then cDNA was synthesized using the GeneChip Whole Transcript (WT) Amplification kit as described by the manufacturer. The sense cDNA was then fragmented and biotin‐labeled with TdT using the GeneChip WT Terminal labeling kit. Approximately 5.5 µg of labeled DNA target was hybridized to the Affymetrix GeneChip Array at 45 °C for 16 h. Hybridized arrays were washed and stained on a GeneChip Fluidics Station 450 and scanned on a GCS3000 Scanner. Array data export processing and analysis were performed using Affymetrix GeneChip Command Console Software. Raw data sets were combined and normalized with Signal Space Transformation‐Robust Multichip Analysis method (SST‐RMA). It performed GSEA (ver4.2.2) of transcriptomic profiles for functional and pathway enrichment analysis. The gene sets were obtained from the Broad MSigDB (Hallmark, KEGG and WP) or extracted from published works (adult murine SMG cell marker genes, Ferroptosis driver genes)^[^ ^51^, ^52^ ^]^ (Table S1, Supporting Information). The analysis results are available in Table S2 (Supporting Information). Meanwhile, DEGs between control‐ and OVX‐SGs were identified with SST‐RMA method. Statistical significance of the expression data was determined by independent t‐test and FDR controlled by adjusting p‐value using Benjamini–Hochberg algorithm. Genes were listed whose |fold change(FC)| was ≥1.5 and the p‐value was ≤0.05 as DEGs. The PPI network within DEGs was constructed using STRING (ver. 11.5)^[^ ^53^ ^]^ with setting of Network type: Full; meaning of network edges: confidence; minimum required interaction score: 0.4. The entire network was exported to Cytoscape (ver.3.9.1) then cluster modules within the network were identified using MCODE plugin.^[^ ^54^ ^]^ The parameters used for cluster selection parameters were as follows: degree cutoff, 2; node score cutoff, 0.2; and K‐core, 2; Maximum depth, 100. Among the modules, Top three modules within upregulated DEGs were further analyzed using the ClueGO plugin (v2.5.4)^[^ ^55^ ^]^ to find enriched key pathways with hub genes. The configuration for generating functionally‐grouped annotated networks included the following settings: Gene ontology (GO) terms were from “GO Biological Processes”, and only pathways with a P‐value below 0.05 were explored. The GO tree interval levels ranged from 3 to 6, with a minimum of three genes and 4% of the gene population per cluster. The minimum k‐score was set to 0.4, while all remaining parameters were set to default.

### Culture of SGOs from Mouse and Human SMG

An advanced SGO culture system was established by adapting currently‐reported methods.^[^ ^50^, ^56^ ^]^ SMGs were enzymatically dissociated with digestion solution for 20 min at 300 rpm, 37 °C. The composition of digestion solution is: Collagenase II (0.6 mg mL^−1^; Gibco, Waltham, MA), hyaluronidase (1 mg mL^−1^; Sigma), CaCl_2_ (50 mm; Sigma) in HBSS (Gibco). After the initial incubation, the supernatant was discarded, and the remaining tissue fragments were dissociated through 2 to 3 additional cycles until complete digestion was achieved. The supernatant containing the isolated SG cells was filtered through a 100 µm cell strainer (BD Biosciences, San Jose, NJ) and centrifuged at 400 g for 5 min. The cell pellet was resuspended in 400 µL of EM containing 5% growth factor‐reduced Matrigel (Corning, Corning, NY) and seeded at a density of 3 × 10^4^ cells per well onto ultra‐low attachment 24 well plates (Sigma) to facilitate suspension culture. Growth media contains 1X B27 (Gibco), 1X N2 (Gibco), Nicotinamide (10 mm mL^−1^; Sigma), EGF (100 ng mL^−1^; Peprotech), Noggin (100 ng mL^−1^; Peprotech), bFGF (100 ng mL^−1^; Peprotech), SB431542 (SB; 10 µm; Cayman) in DMEM/F12 (Gibco). Wnt3a (100 ng mL^−1^; Peprotech) was added to growth media at early passages (P0–P2) to enhance stability, while SB was omitted for TGFβ2 treatment experiments. Half of the media was replenished on day 3, and expanded SGOs were dissociated using TrypLE (Gibco) on day 7 for passaging.

### Analysis of Self‐Renewal Capacity and Viability of SGOs

The proliferative potential of SGOs was analyzed by calculating the cumulative population doubling level (CPDL) using the following formula: ln(Nf/Ni)/ln2 (Ni = the initial cell count numbers at plating, Nf = the final cell count numbers at harvesting, ln = natural log). OFE was determined by the ratio of the total SGO number at day 7 to the initial plating cell number. To assess the viability of SGOs with dead cell labeling, PI staining solution (100 µg mL^−1^; Sigma) was directly added into the culture media and incubated for 15 min. SGO images were captured with either EVOS FL microscope (Thermo) or BioTek Cytation 5 imaging multi‐mode reader (Agilent, Santa Clara, CA).

### In Vitro Chemical Treatment

To evaluate the impact of TGFβ2 on SGO growth, TGFβ2 (Peprotech) was treated to SGOs at day 0 (seeding day) or day 4 then size, number and OFE of SGOs were assessed at day 7. For the rest of the experiment, SGOs were treated with TGFβ2 and other chemicals at day 4 then analyzed 24‐ or 48 h later as indicated. Estradiol (50 nm; Cayman) or SB was co‐treated to inhibit SMAD activation. Z‐VAD‐FMK (10 µm; Cayman) was applied to suppress apoptosis, while Ferrostatin‐1 (1 µm; Cayman) and DFO (10 nM; Cayman) were used as ferroptosis inhibitors. Autophagy initiation was blocked by 3MA treatment (2.5 mM; Medchem Express).

### Knockdown of TβRIII with Lentivirus

Scrambled‐ and TβRIII‐targeted lentiviral shRNA expression vectors with predesigned sequences including puromycin‐resistant gene were purchased from VectorBuilder (Chicago, IL) and lentivirus packaging was conducted with Lenti‐X Packaging Single Shots according to manufacturer's instruction (Takara, Japan). For the lentiviral transfection, SGOs were dissociated with TrypLE as subculture procedure and mixed with 5 MOI of lentiviral particle then centrifuged at 600 g, 32 °C for 30 min. After stabilization for 3 h at 37 °C, cells were seeded to generate organoids. 4 days later, puromycin (1 µg mL^−1^; Gibco) was added to culture media for 48 h to select lentivirus‐transfected SGOs. Knockdown efficiency was analyzed by WB.

### Isolation of Murine SGMCs

SGMCs were cultured as previously reported with some modifications.^[^ ^57^ ^]^ Briefly, murine SMGs were incubated in digestion solution consisting of collagenase type IV (2 mg mL^−1^; Worthington) and CaCl_2_ (2 mm; Sigma) in HBSS for 45 min at 37 °C. Following the filtration through a 70µm strainer (BD), the isolated cells were washed with PBS and seeded at 5 × 10^4^ cells cm^−2^ in DMEM/F12 supplemented with Non‐essential amino acid and 10% of FBS (all from Gibco). The media was changed every 2 to 3 days and subculture was conducted on day 7. SGMCs from passages 2 and 3 were used for further experiments.

### SGO Co‐Culture with SGMCs or SGMC‐Derived CM

For the direct co‐culture with SGOs and SGMCs, SGMCs were seeded at a density of 2 × 10^4^ cells cm^−2^ in a 24‐well plate and cultured in SGO expansion media. After 24 h, EGFP‐expressing SGOs (cultured for 5 days, n = 50) were seeded onto the SGMC layer and co‐cultured for an additional 24 h. In addition, SGOs were cultured with CM derived from SGMCs. To prepare SGMC‐CM, SGMCs were cultured with SGO expansion media for 48 h, and their CM was harvested by passing through 0.2 µm membrane filter. The collected CM was then stored at −80 °C until further use.

### qPCR Analysis

To extract RNA from tissues, freshly isolated SMGs were mechanically dissociated in NucleoZOL (Macherey–Nagel, Germany) as manufacturer's instruction. RNA from in vitro samples was extracted using the RNeasy mini kit (Qiagen, Germany). cDNA was synthesized from 1µg (tissues) or 100 ng (cells) of RNA using 5X RT master mix (Toyobo, Japan) and qPCR was performed using the specific primers and SYBR Green PCR Master Mix (Thermo) with ABI System 7500 (Applied Biosystem, Carlsbad, CA). The primer sequences are indicated in **Table** **1**. The absolute values of each mRNA were determined by quantifying their relative levels using GAPDH as an internal control.

**Table 1:** qPCR Primer sequences.

Gene name	Forward Primer	Reverse primer
*Alox12*	CTCTTGTCATGCTGAGGATGGAC	AAGAGCCAGGCAAGTGGAGGAT
*Amy1a*	TCACACGGGTGATGTCAAGT	CATTGCCACAAGTGCTGTCT
*Chrm1*	TGGTGTGTTCTTCCTTGGAC	ACCCAGGAAGAGCTGATGTT
*Chrm3*	GGTTGGTTTCCTTCGTTCTCTGG	GGAGAGGAACTGGATGTAGCAC
*Cnn1*	ACAAGAGCGGAGATTTGAGCCG	TCATAGAGGTGACGCCGTGTAC
*Duox2*	GAGAAAGGCTGTGACCAAGCAG	TCACGCACTTGCTGGGATGAGT
*Fbxw7*	CGAGACTTCATCTCCTTGCTTCC	CCAGAGAAGGTTATCCTCAGCC
*Fth1*	GCCGAGAAACTGATGAAGCTGC	GCACACTCCATTGCATTCAGCC
*Gapdh*	GGAAGGGCTCATGACCAC	GCAGGGATGATGTTCTGG
*Ireb2*	AGAAACGGACCTGCTCTTCCCA	CCTCTGTCTCAATGCCACCAAC
*Ki67*	CCTTTGCTGTCCCCGAAGA	GGCTTCTCATCTGTTGCTTCCT
*Ck* *14*	GACTTCCGGACCAAGTTTGA	CCTTGAGGCTCTCAATCTGC
*Ck18*	TGACTGTGGAAGTGGATGCC	GTTCCTCGCGGTTCTTCTGA
*Ck* *5*	TCCTCCAGGAACCATCATGTCT	GGGACACCGAGCTGAAGCT
*Ck8*	ATCGAGATCACCACCTACCG	CTGAAGCCAGGGCTAGTGAG
*Prol1*	GATGTGCCCTCCAGGAACTA	GGTTGCATTTGGTTTCGTTT
*Sparc*	CACCTGGACTACATCGGACCAT	CTGCTTCTCAGTGAGGAGGTTG
*Tgfb1*	TGATACGCCTGAGTGGCTGTCT	CACAAGAGCAGTGAGCGCTGAA
*Tgfb2*	TTGTTGCCCTCCTACAGACTGG	GTAAAGAGGGCGAAGGCAGCAA
*Tgfb3*	AAGCAGCGCTACATAGGTGGCA	GGCTGAAAGGTGTGACATGGAC
*Thbs1*	GGTAGCTGGAAATGTGGTGCGT	GCACCGATGTTCTCCGTTGTGA

### Immunofluorescence Analysis

SMG tissues were perfused and frozen for cryosection following established protocol. SG sections with a thickness of 10 µm were washed with PBS and incubated in a blocking solution containing 5% normal goat serum (Vector Laboratories, Canada) for 2 h at RT, followed by overnight incubation in primary Ab solutions at 4 °C. The next day, sections were washed with PBS then secondary Ab solutions were applied. Staining for F‐actin and nuclei was performed using Phalloidin and Hoechst, respectively. To obtain SGO sections, SGOs were collected by centrifugation and fixed with 4% PFA for 30 min at RT. Following the fixation, the organoids were transferred to a 30% sucrose solution and incubated for 24 h at 4 °C. The sedimented SGOs were subsequently embedded in the OCT compound (Sakura, Japan) and stored at −80 °C until cryosectioning. SGO sections of 5 µm‐thickness were subjected to immunostaining following the same protocol as described for the SG tissue sections. Images were captured using an LSM 700 Zeiss confocal microscope (Zeiss) or BioTek Cytation 5 imaging multi‐mode reader (Agilent). Quantification of positive signals was conducted using an automated analysis software in Cytation 5. The information of primary‐ and secondary Abs used in this study is available in **Table** **2**.

**Table 2:** Antibody information.

Antibody	Application	Company	Cat.no	Host (Dilution)
AQP5	IHC, ICC	Alomone labs	AQP‐005	R (1:250)
βACTIN	WB	Cell Signaling	#3700	M (1:2000)
CK14	IHC	Biolegend	#905303	R (1:500)
CK7	IHC	Abcam	ab181598	R (1:500)
FTH1	WB	Cell Signaling	#4393	R (1:1000)
FTH1	IHC	Abcam	Ab65080	R (1:500)
GAPDH	WB	Cell Signaling	#5174	M (1:2000)
GPX4	WB	Cell Signaling	#52455	R (1:1000)
KEAP1	WB	Cell Signaling	#8047	R (1:1000)
LC3I/II	WB	Cell Signaling	#12741	R (1:1000)
MDA	IHC, WB	Abcam	ab27642	R (1:500)
NKCC1	IHC	Santa cruz	sc‐514774	M (1:100)
NRF2	WB	Cell Signaling	#12721	R (1:1000)
p62	WB	Proteintech	#18420	R (1:1000)
pSMAD2	WB	Cell Signaling	#3108	R (1:1000)
SMAD2	WB	Cell Signaling	#5339	R (1:1000)
TFR1	IHC, ICC, WB	Invitrogen	#13‐6800	R (1:500)
TGFβ2	IHC, ICC, WB	Abclonal	#A3640	R (1:500)
TGFβ2	IHC, ICC	Abcam	Ab36495	M (1:500)
TβRIII	WB, flow cytometry	Santa cruz	Sc‐74511	M (1:1000)
THBS1	ICC	Santa cruz	sc‐59887	M (1:500)
xCT	WB	Abclonal	#A15604	R (1:1000)

### Flow Cytometry Analysis

SGOs were harvested by brief centrifugation and were dissociated into single cells using TryPLE (Gibco) at 37 °C for 15 min. The cells were blocked with 3% bovine serum albumin solution in PBS and then incubated with CD117/c‐kit‐FITC, CD49f‐PE, CD326/Epcam‐APC, CD24‐APC and CD29‐FITC (all from BD Biosciences) at a ratio of 1:100 for 60 min at 4 °C. Following incubation, samples were washed twice with PBS then proceeded to analysis with FACS Canto II or Accuri C6 Plus flow cytometer (BD Biosciences). Data were processed using FlowJo software (Tree Star Inc., Ashland, OR, USA). The proportion of proliferating cells within the SGOs was evaluated using a Click‐iT EdU Flow Cytometry Assay Kit (Thermo) according to the manufacturer`s instructions. Prior to analysis, the EdU solution (10 µm) was added directly to the culture media for 3 h. The localization of TfR1 in SGOs was also evaluated via flow cytometry. After the designated chemical treatment, SGOs were dissociated into single cells as above and then incubated in a TfR1 Ab solution (1:500, Thermo) for 2 h at 4 °C. After the washing step, cells were incubated in the Alexa fluor 488‐conjugated secondary Ab solution for 30 min at RT then their fluorescence was analyzed.

### WB Analysis

SG tissues and SGOs were lysed with PRO‐PREP protein extraction solution (iNtRON Biotechnology, South Korea), and proteins were extracted according to the manufacturer's instruction. The remaining procedure was performed as established WB protocol. The blot images were captured using a LAS4000 imaging system (GE Healthcare, Chicago, IL), while the band intensities were measured by the NIH Image J software. The information of primary‐ and secondary Abs is available in Table 2.

### Lipid Peroxidation Assay of SGOs

SGOs cultured for 5 days (*n* = 80) were mixed with Matrigel at a ratio of 3:1 and seeded onto 96 well plates in a dome shape. After polymerization, media were added to each well accompanied by various chemical treatments as described in the result section. On the following day, Cell‐based Lipid Peroxidation Assay Kit (Abcam) was applied to evaluate the lipid peroxidation levels in SGOs. After the assay, Hoechst staining was performed to indicate individual SGO. The images were captured using a BioTek Cytation 5 (Agilent), and the ratio between red to green fluorescence of each organoid was calculated by the automated analysis software.

### MDA Measurement in the Tissue Samples

Evaluation of in vivo lipid peroxidation level was conducted using MDA Assay Kit (Dojindo, Japan) that detects one of the end products of lipid peroxides MDA by colorimetric methods. Freshly isolated SGs were homogenized on ice in an antioxidant PBS solution (included in the kit) and centrifuged at 10 000 g to collect the supernatant. Measurement of MDA was conducted as described in the instructions. Absorbance at 532 nm was detected using a microplate reader Synergy HTX (Agilent).

### Statistics

All statistical comparisons were determined by Student's t‐test or one‐way ANOVA, followed by Dunnett's multiple comparisons test using GraphPad Prism software ver 10.0.0 (GraphPad, San Diego, CA) as indicated in the figure legends. Results were presented as means ± SEM and P‐values under 0.05 were considered as significant.

### Study Approval

The animal experiment was approved by the IACUC of Pusan National University (PNU‐2019‐2471, L2023‐013‐A1C0). For the primary culture of human SGOs, the SMGs of female patients who underwent SMG resection surgery due to various diseases (including sialolithiasis, benign tumors and carcinoma) were collected with the approval of the IRB of Pusan National University Hospital (IRB No. H‐1907‐025‐080) and acquisition of informed consents. This study also conducted immunohistochemical analysis using paraffin‐embedded female SMG sections, which were assigned random numbers and approved by the IRB of Pusan National University Hospital (IRB No. H‐1910‐031‐084). All donor information was blinded except for age; a total of three SMG slides from premenopausal women (ages 26 and 32) and postmenopausal women (ages 62, 69, and 75) were used. Only tissue specimens verified as normal via histological analysis were utilized for experiments.

### Data Availability

All microarray data were deposited in a Minimum Information About a Microarray Experiment (MIAME)‐compliant public database under the accessions GSE241395.

## Conflict of Interest

The authors declare no conflict of interest.

## Author Contributions

S.‐J.O. and Y.Y.S. contributed equally to this work. As the first author, S.‐J.O. and Y.Y.S. performed most experiments, analyzed the data and wrote the draft of manuscript. J.‐S.A. and H.‐J.P. supported OVX surgery and management of mice. P.T.N. and D.K.N. supported saliva collection. S.S.P. and M.‐J.K. supported in vitro experiments. T.‐H.S., B.‐C.L., and J.‐H.J. conducted evaluation and quantification analysis of image data. W.K.K. provided guidance on SGO culture setting. J.‐M.O., D.J., and Y.H.K. performed bioinformatics analysis. J.M.K. and E.‐S.S. conducted IHC on human samples. E.‐W.L. wrote the manuscript. B.‐J.L., Y.S., and H.‐S.K. designed the research, interpreted the data, wrote and edited the manuscript with feedback from all authors.

## Supporting information

Supporting Information

## Data Availability

The data that support the findings of this study are available from the corresponding author upon reasonable request.
