# Supplementary material for: TGFβ2‐Driven Ferritin Degradation and Subsequent Ferroptosis Underlie Salivary Gland Dysfunction in Postmenopausal Conditions
Source: Adv Sci (Weinh). 2024 Nov 1;11(47):2400660. doi: 10.1002/advs.202400660 (PMC11653620; doi:10.1002/advs.202400660)
Supplement: Supplementary file 1 — Supporting Information [file ADVS-11-2400660-s001.docx]

Supporting Information

Title

TGFβ2-Driven Ferritin Degradation and Subsequent Ferroptosis Underlie Salivary Gland Dysfunction in Postmenopausal Conditions

*Su-Jeong Oh, Ye Young Shin, Ji-Su Ahn, Hee-Jeong Park, Min-Jung Kang, Tae-Hoon Shin, Byung-Chul Lee, Won Kyu Kim, Jung-Min Oh, Dongjun Lee, Yun Hak Kim, Ji Min Kim, Eui-Suk Sung, Eun-Woo Lee, Jee-Heon Jeong, Byung-Joo Lee*, Yoojin Seo*, and Hyung-Sik Kim**


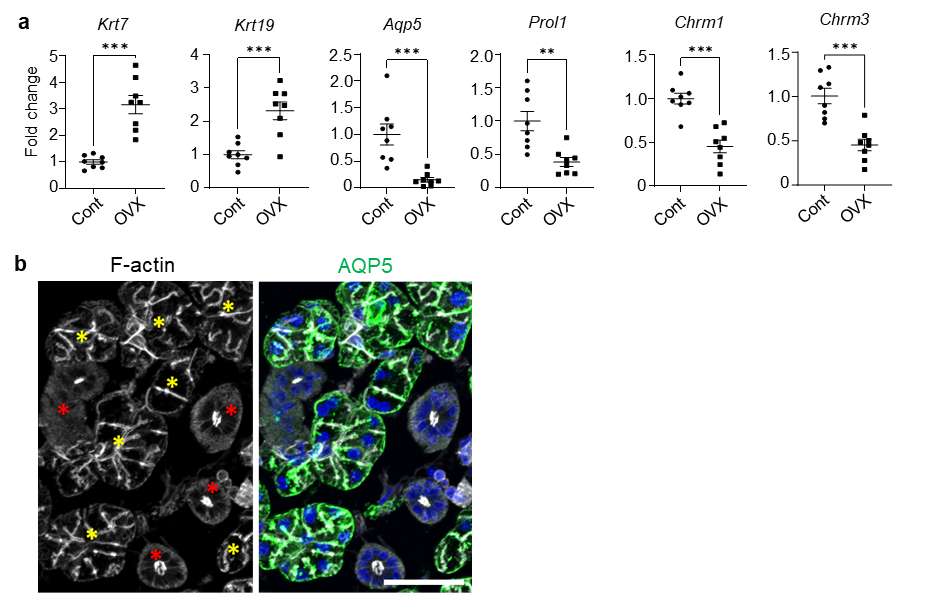


**Figure S1.** OVX changes duct/acini markers in mice. (a) qPCR was conducted for duct (Krt7, Krt19) and acini (Aqp5, Prol1, Chrm1, Chrm3) markers with Cont- and OVX-SGs (n=8 for each). (b) Representative F-actin stained SG images with (right) or without (left) AQP5 labeling. Acini (yellow asterisks) and duct structures (red asterisks) can be differentiated by the F-actin stained pattern. Scale bar = 40 μm. Data are shown as the mean ± SEM and compared by unpaired t-test. **P < 0.01, ***P < 0.001.


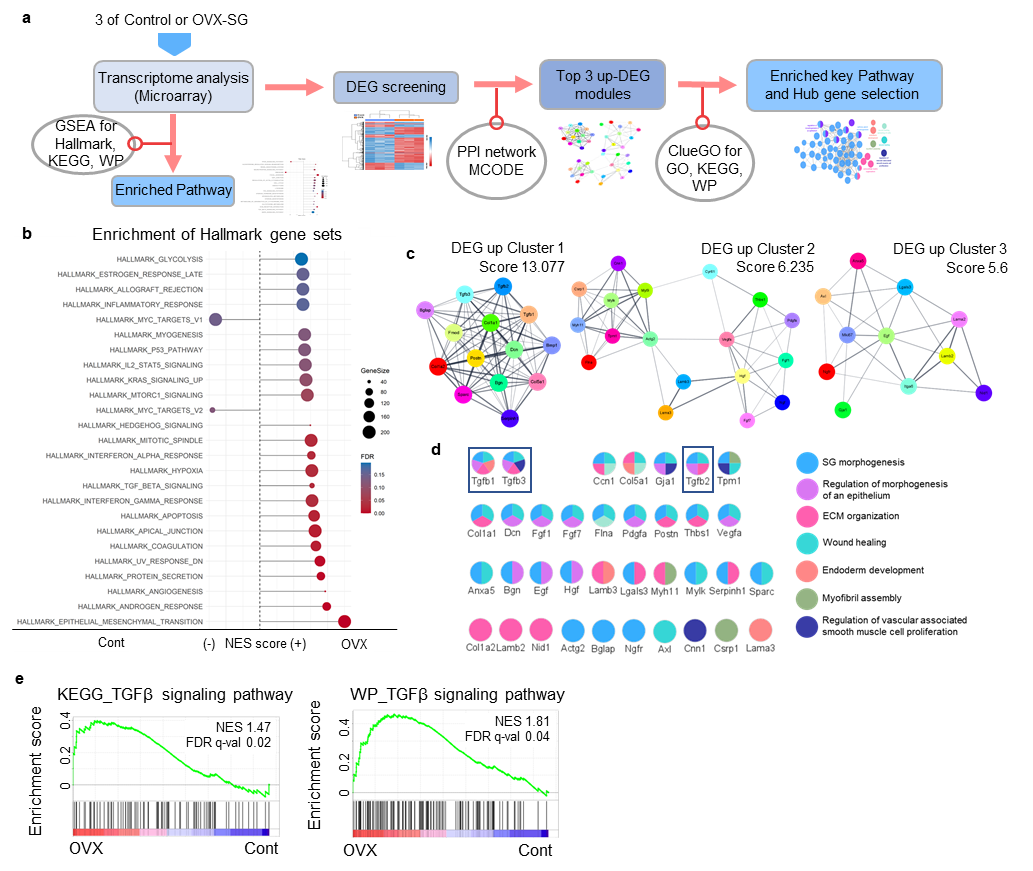


**Figure S2.** Microarray-based functional enrichment analysis of Cont- and OVX-SGs. (a) Workflow of the bioinformatics analysis. (b) GSEA of the hallmark gene sets comparing Cont- and OVX-SGs. (c) Identification of the top three modules from the upregulated DEGs in OVX-SGs. (d) Enriched GO-BP terms-associated genes in the Top 3 modules of OVX-SGs. (e) GSEA comparing Cont- and OVX-SGs using various TGFβ signaling gene signatures extracted from KEGG and Wiki Pathways. Data are shown as the mean ± SEM and compared by unpaired t-test. *P < 0.05, **P < 0.01.


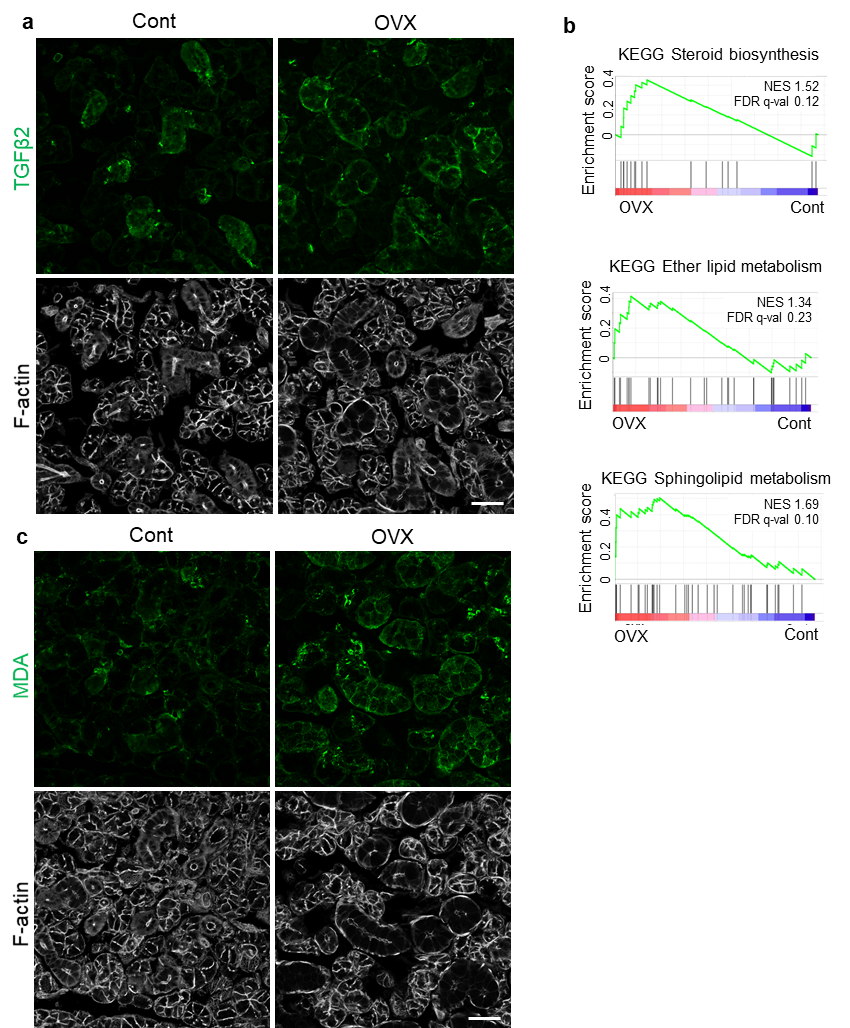


**Figure S3.** Transcriptome profiling reveals enrichment of TGFβ signaling and ferroptosis pathways in OVX-SG. (a) Individual images from Figure 2f prior to merging, showing separate staining details. (b) GSEA of Cont- and OVX-SGs using lipid metabolism-related gene signatures. (c) Individual images from Figure 2j prior to merging, displaying detailed staining pattern. Scale bar = 40 μm.


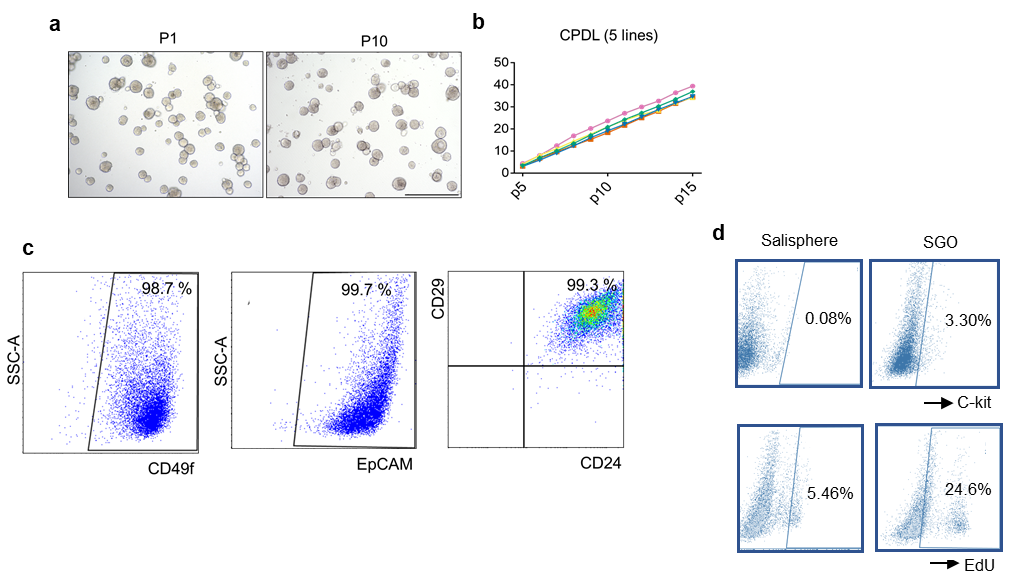


**Figure S4.** Establishment of expansion conditions for murine SGOs. (a) Typical morphology of expanding SGOs. (b) CPDL of established SGOs (*n*=5) from P5 to P15. (c) Flow cytometric analysis of SGOs for epithelial cell markers and SGSC marker, c-kit. (d) Flow cytometry analysis of c-kit and EdU^+^ cells within the salispheres and SGOs. Scale bar = 500 μm.


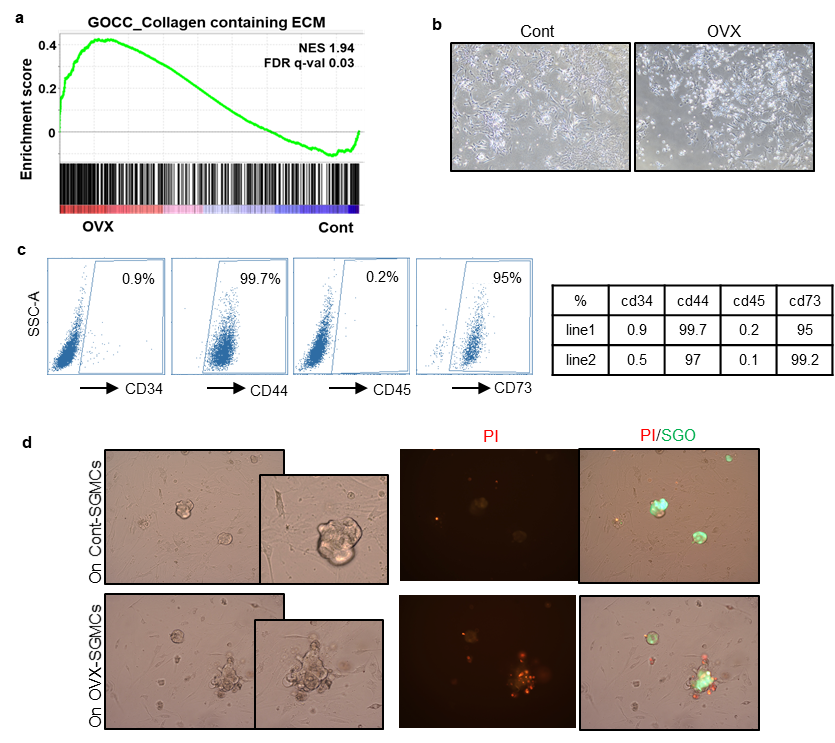


Figure S5. Characterization SGMCs and of co-culture of SGOs with SGMCs. (a) GSEA of collagen-containing ECM from GO-CC using the entire gene between Cont- and OVX-SGs. (b) Representative growing morphologies of SGMCs (at day 7) derived from Cont- and OVX-mice. (c) Flow cytometry-based validation of the MSC-associated CD marker expression in SGMCs. (d) Representative images of PI-labeled dying SGOs cultured on a layer of SGMCs. Scale bar = 500 μm.


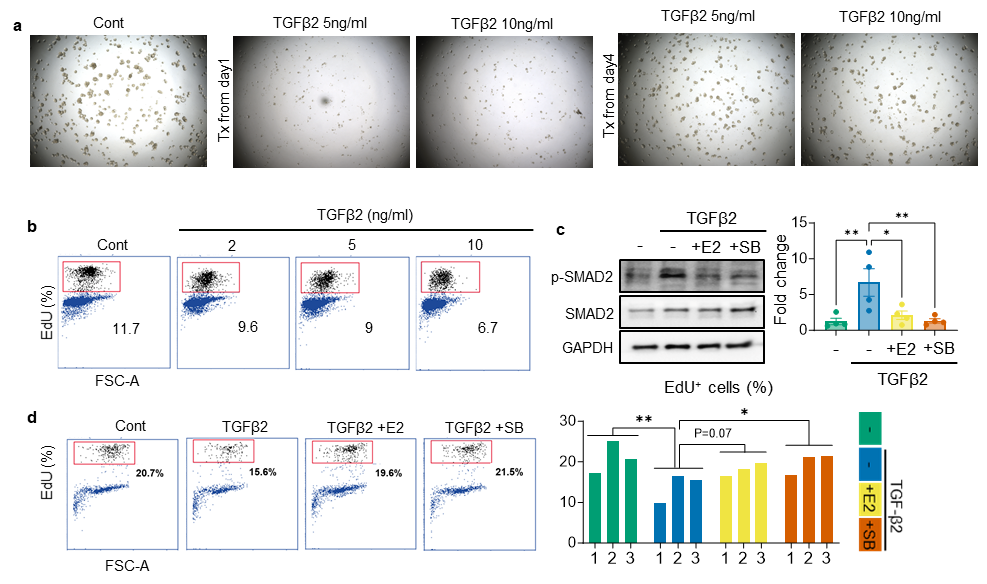


Figure S6. The negative impact of TGFβ2 on SGO growth. (a) Representative images of SGOs following TGFβ2 treatment under the indicated treatment conditions. (b) Flow cytometry analysis of EdU^+^ cells within control- and TGFβ2 treated SGOs. (c) WB analysis demonstrating that SMAD2 phosphorylation, induced by TGFβ2 treatment, was suppressed by the administration of E2 and SB. (d) Flow cytometry analysis and quantification of EdU^+^ cells in SGOs cultured with indicated treatment. At least 3 lines of SGOs were used for all experiments. Scale bar = 500 μm. Data are shown as the mean ± SEM and compared by one-way ANOVA with Dunnett`s multiple comparisons test. *P < 0.05, **P < 0.01, ***P < 0.001.


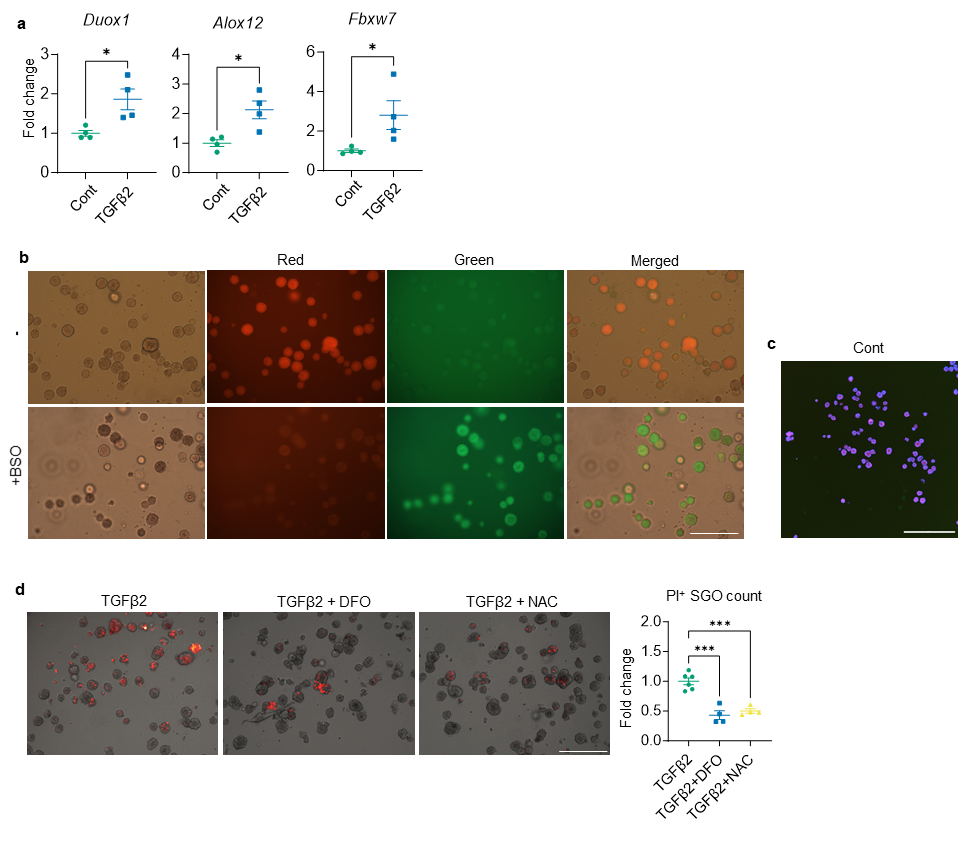


Figure S7. TGFβ2 increases lipid peroxidation and ferroptosis in SGOs. (A) qPCR results showing the mRNA expression of ferroptosis-associated markers in cont- and TGFB2-treated SGOs. (b) Representative bright field and fluorescence images of SGOs under lipid a peroxidation assay, with ferroptosis inducer BSO-treated SGOs serving as a positive control. (c) Representative fluorescence image of control SGOs under lipid a peroxidation assay. Most organoids exhibit a purple color, resulting from the combination of blue colored-Hoechst staining and red fluorescence indicating an unperoxidized state. At least 3 lines of SGOs were used for all experiments. Scale bar = 500 μm (b, d) and 1 mm (c). Data are shown as the mean ± SEM and compared by unpaired t-test (a) or one-way ANOVA with Dunnett`s multiple comparisons tests (d). *P < 0.05, ***P < 0.001.


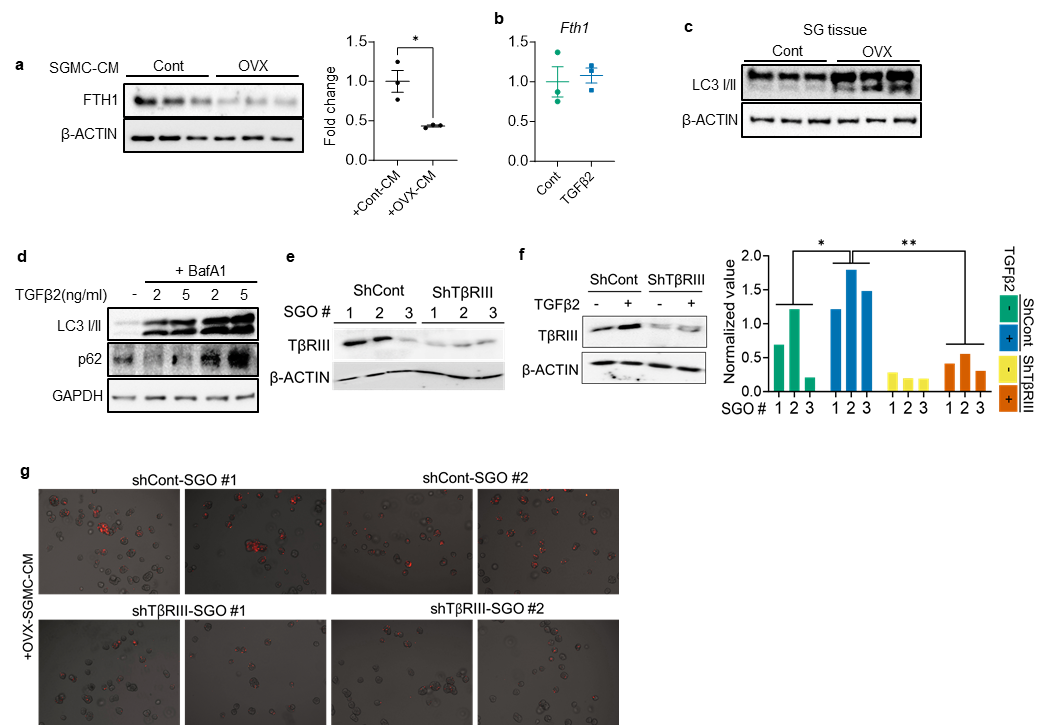


**Figure S8.** Degradation of FTH1 underlies TGFβ2-mediated ferroptosis in SGOs. (a) WB analysis of FTH1 in SGOs cultured with CM of SGMCs derived from Cont- and OVX-mice. (b) mRNA expression levels of FTH1 in SGOs with or without TGFβ2 treatment. (c, d) Immunoblot analysis for autophagy markers in SG tissues (c) and SGOs exposed to TGFB2, 3MA and BafA1 (d). (e) WB results confirming the knockdown of TβRIII expression in shTβRIII-SGOs. (f) Following TGFβ2 exposure, changes in TβRIII levels in shCont- and shTβRIII-SGOs were evaluated using WB. (g) Representative PI-stained images of shCont- and shTβRIII-SGOs cultured with CM of SGMCs derived from Cont- and OVX-mice. Data are shown as the mean ± SEM and compared by unpaired t-test (a, b) or one-way ANOVA with Dunnett`s multiple comparisons tests (f). *P < 0.05 and **P < 0.01.


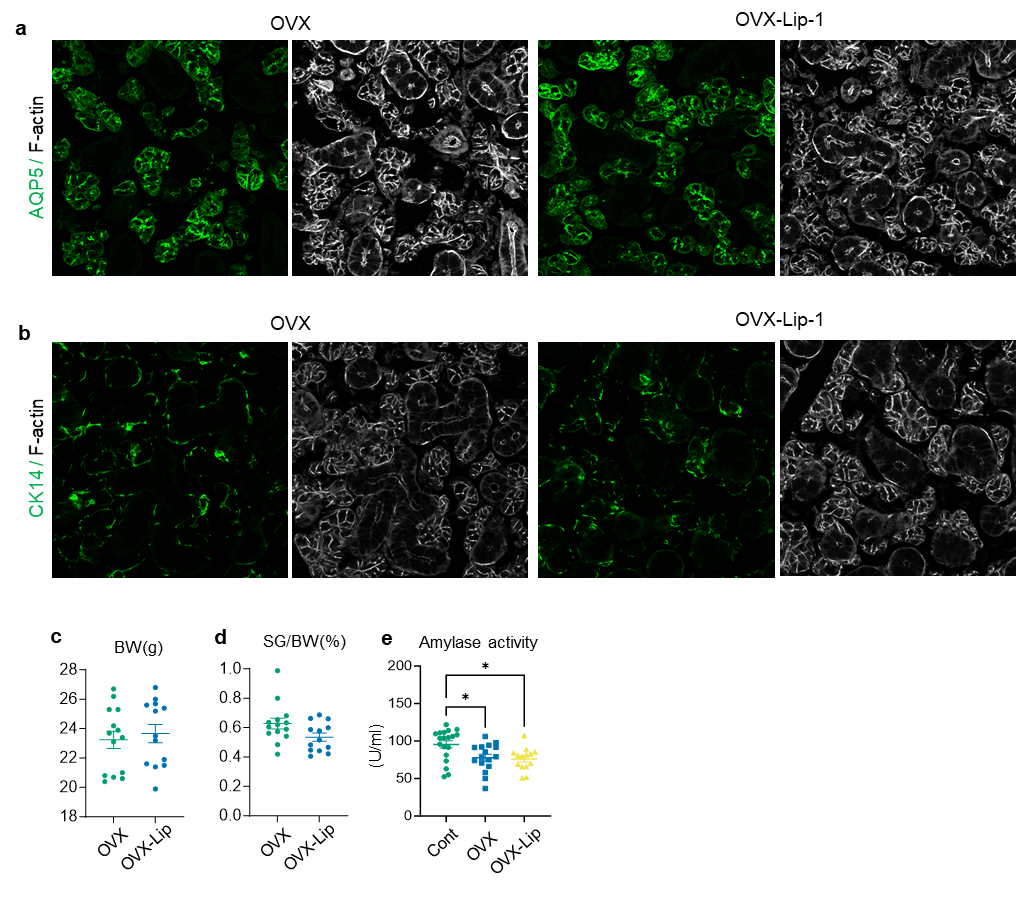


**Figure S9.** In vivo administration of ferroptosis inhibitor can contribute to SG recovery in OVX mice. (a, b) Individual images from Figure 7e (a) and 7f (b) prior to merging, showing separate staining details. (c, d) Effect of Lip treatment on BW (b) and SG weight (c) of OVX mice. (d) The amylase activities of collected saliva were determined. Data are shown as the mean ± SEM and compared by one-way ANOVA with Dunnett`s multiple comparisons tests. *P < 0.05.


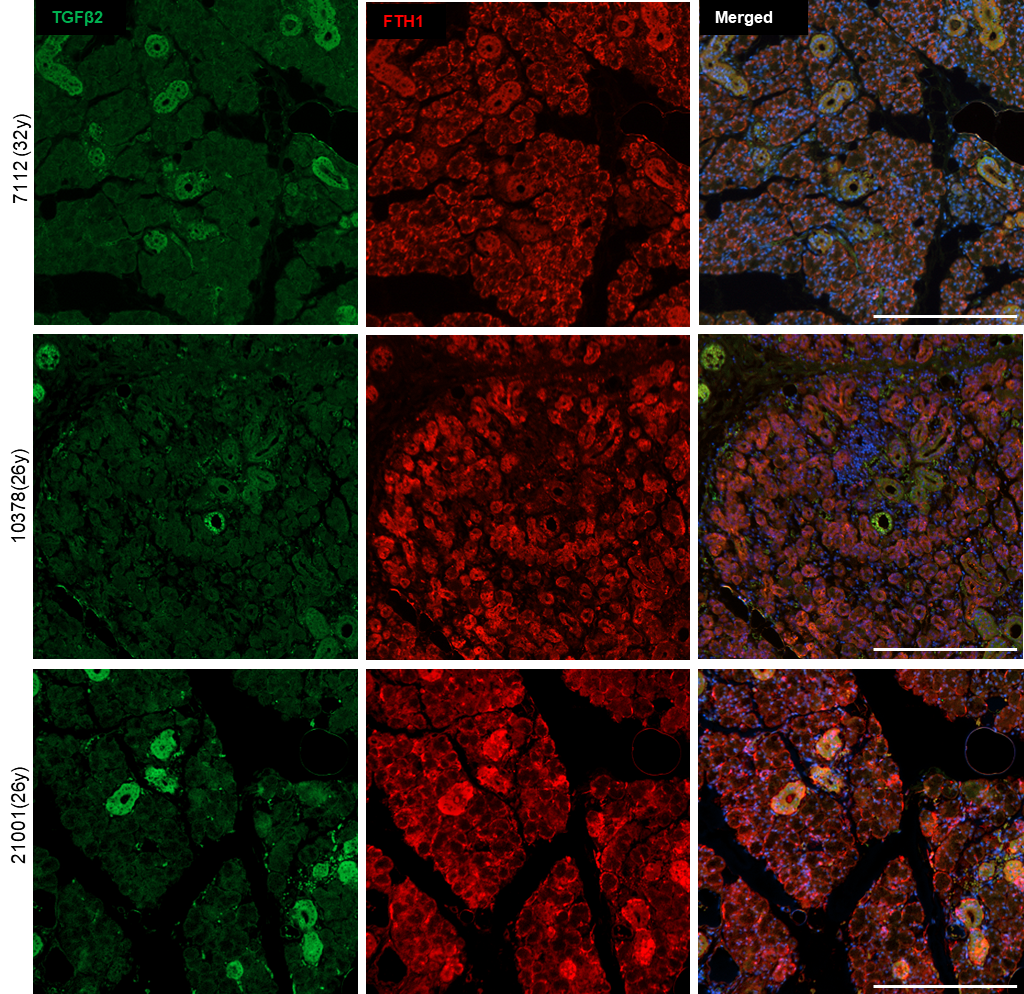


**Figure S10.** Representative SMG images of three premenopausal women stained for FTH1 (red) and TGFβ2 (green). Scale bar = 300 μm.


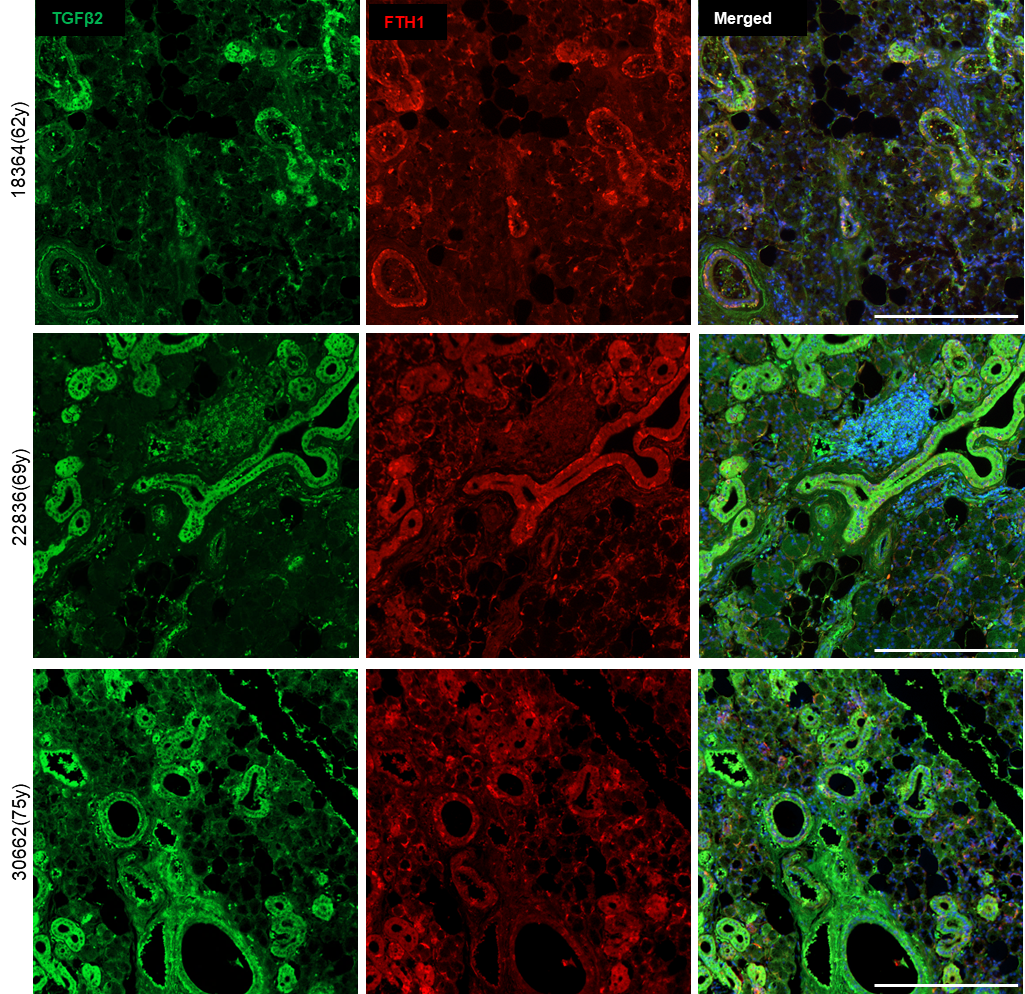


**Figure S11.** Representative SMG images of three postmenopausal women stained for FTH1 (red) and TGFβ2 (green). Scale bar = 300 μm.
